# Crosstalk between cancer-associated fibroblasts and immune cells in the tumor microenvironment: new findings and future perspectives

**DOI:** 10.1186/s12943-021-01428-1

**Published:** 2021-10-11

**Authors:** Xiaoqi Mao, Jin Xu, Wei Wang, Chen Liang, Jie Hua, Jiang Liu, Bo Zhang, Qingcai Meng, Xianjun Yu, Si Shi

**Affiliations:** 1 Department of Pancreatic Surgery, Fudan University Shanghai Cancer Center, No. 270 Dong’An Road, Xuhui District, Shanghai, 200032 China; 2 Department of Oncology, Shanghai Medical College, Fudan University, Shanghai, 200032 China; 3 Shanghai Pancreatic Cancer Institute, Shanghai, 200032 China; 4 Pancreatic Cancer Institute, Fudan University, Shanghai, 200032 China

**Keywords:** Cancer-associated fibroblasts, Tumor-infiltrating immune cells, Tumor microenvironment, Tumor immune microenvironment, Cell–cell interaction, Immune suppression, CAF-targeted therapy, Cancer

## Abstract

Cancer-associated fibroblasts (CAFs), a stromal cell population with cell-of-origin, phenotypic and functional heterogeneity, are the most essential components of the tumor microenvironment (TME). Through multiple pathways, activated CAFs can promote tumor growth, angiogenesis, invasion and metastasis, along with extracellular matrix (ECM) remodeling and even chemoresistance. Numerous previous studies have confirmed the critical role of the interaction between CAFs and tumor cells in tumorigenesis and development. However, recently, the mutual effects of CAFs and the tumor immune microenvironment (TIME) have been identified as another key factor in promoting tumor progression. The TIME mainly consists of distinct immune cell populations in tumor islets and is highly associated with the antitumor immunological state in the TME. CAFs interact with tumor-infiltrating immune cells as well as other immune components within the TIME via the secretion of various cytokines, growth factors, chemokines, exosomes and other effector molecules, consequently shaping an immunosuppressive TME that enables cancer cells to evade surveillance of the immune system. In-depth studies of CAFs and immune microenvironment interactions, particularly the complicated mechanisms connecting CAFs with immune cells, might provide novel strategies for subsequent targeted immunotherapies. Herein, we shed light on recent advances regarding the direct and indirect crosstalk between CAFs and infiltrating immune cells and further summarize the possible immunoinhibitory mechanisms induced by CAFs in the TME. In addition, we present current related CAF-targeting immunotherapies and briefly describe some future perspectives on CAF research in the end.

## Introduction

In recent years, the tumor microenvironment (TME) has received increasing attention due to its crucial roles in tumor immune suppression, distant metastasis, local resistance and the targeted therapy response [1–4]. The TME is a highly complicated system mainly composed of tumor cells, infiltrating immune cells (such as macrophages, dendritic cells and lymphocytes), cancer-associated stromal cells (such as cancer-associated fibroblasts (CAFs)), endothelial cells and lipocytes, along with the extracellular matrix (ECM) and multiple signaling molecules [5, 6]. As one of the most important stromal components in the TME, CAFs have shown biological heterogeneity in many aspects, including the cell of origin, phenotype and function [7, 8]. Originating from a variety of cell types, CAFs are characterized by increased expression of markers such as alpha smooth muscle actin (α-SMA), fibroblast activation protein (FAP), fibroblast-specific protein 1 (FSP1), platelet-derived growth factor receptor (PDGFR)-α/β and vimentin [9]. Most of CAF subpopulations usually exhibit cancer-promoting effects, while the discovery of cancer-restraining CAFs (rCAFs), which are reported to exert inhibitory effects on tumor progression, indicates that some subsets are just the opposite [10]. Substantial previous reports have demonstrated that CAFs participate in multiple stages of tumor development through diverse pathways [11–13]. Through bidirectional signaling with tumor cells and other cells mediated by CAF-derived cytokines, chemokines, growth factors and exosomes within the TME, CAFs not only facilitate tumor proliferation but also induce immune evasion of cancer cells [14–16]. Moreover, CAFs are also able to degrade stromal ECM by releasing matrix metalloproteinases (MMPs) while synthesizing new matrix proteins to provide structural support for tumor invasion and angiogenesis [17, 18]. Overall, more specific roles and detailed mechanisms of CAFs in cancer pathogenesis and progression remain to be further explored.

The tumor immune microenvironment (TIME) is a novel proposed concept that has been reported to be closely related to the clinical prognosis of patients with tumors [19]. Distinct immune cell populations, including innate and adaptive immune cells, such as myeloid cells and lymphocytes within the TME, comprise most of the TIME [20, 21]. Notably, the TIME also determines the state of the immune response in the TME, which primarily depends on the composition and activity of infiltrated immune cells, as well as several correlated influencing factors, including the cell surface expression of immune checkpoint molecules and alterations in the associated matrix [20]. Currently, an increasing number of researchers have begun to focus on the immunosuppressive effect of CAFs that is achieved by interactions with TIME components, especially immune cells [22–24]. For instance, CAFs are capable of restricting the recruitment of immune effector cells such as CD8 + T cells into tumor tissues through the secretion of different chemokines [25]. Moreover, the proportions of immunosuppressive cells such as M2-type macrophages, regulatory T (Treg) cells and myeloid-derived suppressor cells (MDSCs), which are modified by CAFs, have been shown to be significantly increased in the TIME, thereby contributing to tumor immune suppression [26–28]. Additionally, some cytokines secreted by activated immune cells, such as interleukin (IL)-1β, can induce the transformation of normal fibroblasts into proinflammatory CAFs and further facilitate the recruitment of inhibitory immune cells and immune suppression in the TME [29]. Undeniably, a deep understanding of the multidimensional interactions between CAFs and infiltrating immune cells within the TME will help us better to determine the immunosuppressive mechanisms induced by CAFs, and further exploration of these interactions will probably identify more potential molecular targets for CAF-targeted therapy.

This review mainly focuses on recent advances in the crosstalk between CAFs and tumor-infiltrating immune cells, immune checkpoint molecules and related ECM alterations in the TME, along with the possible mechanisms of CAF-induced immune suppression according to these interactions. We also describe the current understanding of the origins, activators, heterogeneity and plasticity of CAFs. Finally, we introduce major CAF-based targeted immunotherapeutic strategies that may enhance antitumor immunity in the TME, and present some deficiencies of CAF studies currently existed and several promising research directions in the future.

## Origins and activators of CAFs

**Fig. 1:**
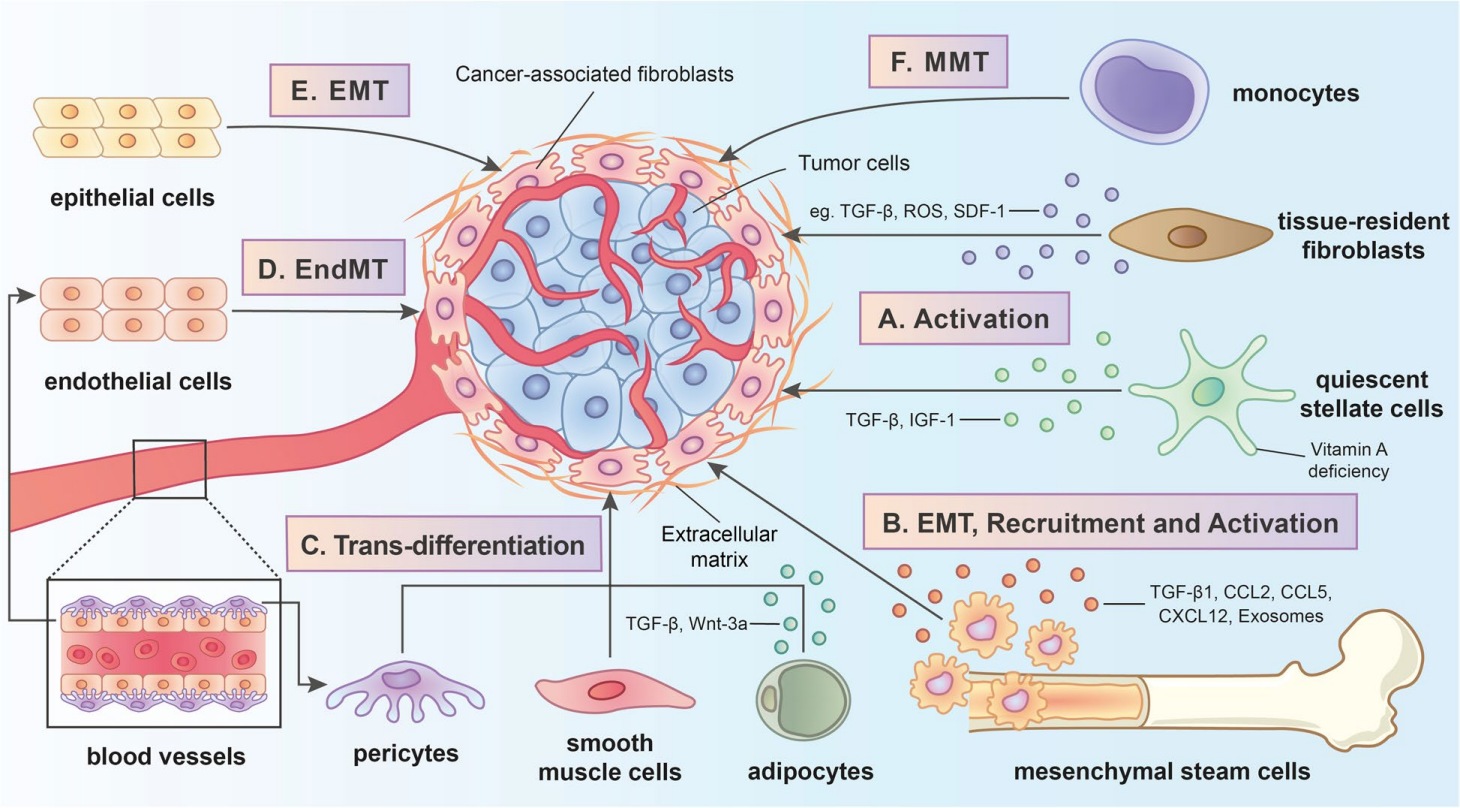
The origins and related activating pathways of cancer-associated fibroblasts (CAFs) in the tumor microenvironment (TME). CAFs are derived from multiple cell types through the following distinct mechanisms: **A** Tissue-resident fibroblasts and quiescent stellate cells are converted into CAFs by the stimulation of modulators including transforming growth factor-beta (TGF-β), hepatocyte growth factor (HGF), platelet-derived growth factor (PDGF), fibroblast growth factor 2 (FGF-2), stromal-derived factor-1 (SDF-1), reactive oxygen species (ROS) and insulin-like growth factor 1 (IGF-1) as well as the deficiency of vitamin A; **B** The trans-differentiation progress of mesenchymal stem cells (MSCs) into CAFs contain epithelial-mesenchymal transition (EMT) along with the recruitment and activation induced by various stimulating molecules such as TGF-β1, C–C chemokine ligand 2 (CCL2), C–C chemokine ligand 5 (CCL5), C-X-C chemokine ligand 12 (CXCL12) and tumor-derived exosomes; **C** Adipocytes together with pericytes and smooth muscle cells can transdifferentiate into CAFs by TGF-β1 and Wnt3a; **D** Endothelial cells are transformed into CAFs through endothelial-to-mesenchymal transition (EndMT); **E** Epithelial cells are transformed into CAFs through epithelial-to-mesenchymal transition (EMT); **F** Monocytes are transformed into CAFs through monocyte-to-myofibroblast trans-differentiation (MMT)

Activated by diverse signaling pathways, CAFs are derived from multiple cells of origin (FIG. 1). The presence of various cellular precursors for CAFs, which might explain why CAFs are a heterogeneous cell population, has been confirmed by a large amount of evidence [8, 30, 31].

Tissue-resident fibroblasts, also termed quiescent fibroblasts, are one of the major sources of CAFs [32, 33]. Tissue-resident fibroblasts in distinct carcinomas are recruited and activated through the stimulation of different modulators [34], including transforming growth factor (TGF)-β [35], hepatocyte growth factor (HGF) [36], platelet-derived growth factor (PDGF) [37], fibroblast growth factor 2 (FGF-2) [37], stromal-derived factor-1 (SDF-1) [38] and reactive oxygen species (ROS) [39, 40]. In some tumor types, stellate cells might be another source of CAFs. For instance, in liver and pancreatic cancer models, quiescent pancreatic stellate cells (PSCs) and hepatic stellate cells (HSCs) express CAF-like surface markers such as α-SMA upon activation with TGF-β and PDGF, which convert them into activated CAFs [41, 42]. Moreover, vitamin A deficiency has been reported to be involved in the activation of PSCs and islet stellate cells (ISCs) [43, 44]. Furthermore, a recent study revealed that the stimulation of insulin-like growth factor 1 (IGF-1) signaling is also critical for HSC activation [45].

A substantial number of reports have indicated that mesenchymal stem cells (MSCs) function as precursors of CAFs [46, 47]. The transformation of bone marrow mesenchymal stem cells (BMSCs) into CAFs might be a multistep and complicated process involving epithelial-mesenchymal transition (EMT), bone marrow-derived progenitors, cell–cell communication and stimulation with various cytokines [48–50]. Effectors stimulating MSC trans-differentiation vary from cancer to cancer. In prostate carcinoma, MSCs transdifferentiate into CAFs through the activation of tumor cell- and stromal cell-secreted TGF-β1, as well as C-X-C chemokine ligand (CXCL) 16 [51, 52]. In breast cancer, bone marrow-derived mesenchymal stem cells (BM-MSCs) are recruited and transformed into distinct CAF subsets through the TGF-β1-mediated osteopontin-myeloid zinc finger 1 (MZF1) pathway [53]. Subsequent studies further confirmed the importance of the TGF-β signaling pathway in the transformation of MSCs into CAFs; for example, the pathway is involved in their metabolic reprogramming toward increased glycolysis [54]. In addition, secreted C–C chemokine ligand (CCL) 2, CCL5 and CXCL12 in the TME are also involved in the recruitment and transformation of MSCs [46, 55]. Notably, multiple internal mechanisms for MSC transformation might exist. For example, the differentiation of BM-MSCs into CAFs induced by cancer cells was reported to primarily depend on the Notch and Akt signaling pathways [56]. However, Peng et al. [57] discovered that tumor-derived GRP78, an endoplasmic reticulum (ER) chaperone, elicits the differentiation of BM-MSCs into CAFs in a TGF-β/SMAD-dependent manner.

Adipocytes, especially white adipocytes [58, 59], are regarded as another cell type within CAF precursors. For instance, human adipose tissue-derived stem cells (HASCs) transdifferentiate into CAFs with a fibroblastic phenotype (α-SMA and tenascin-C expression) upon activation with TGF-β1 [60]. Moreover, research in breast cancer has found that Wnt3a produced by tumor cells mediates the β-catenin-dependent differentiation of adipocytes into adipocyte-derived fibroblasts (ADFs), which express one CAF marker, FSP-1, at high levels [61, 62]. Furthermore, several other potential sources of CAFs have been identified, such as epithelial cells [63, 64], pericytes [65], monocytes [66], endothelial cells [67] and smooth muscle cells [68]. These cells can be activated and differentiate into CAFs through various mechanisms. Through EMT and endothelial-to-mesenchymal transition (EndMT), most epithelial cells and endothelial cells can express a number of fibroblast markers, such as α-SMA and FAP [69, 70]. Peritoneal mesothelial cells, one of the special cell types among epithelial cells, are reported to be converted into CAFs through TGF-β1-induced mesothelial-mesenchymal transition (MMT) [71]. Additionally, monocytes are able to transdifferentiate into myofibroblasts through a process termed monocyte-to-myofibroblast trans-differentiation (MMT), which is induced by ROS through the p38-mitogen-activated protein kinase (MAPK) signaling pathway [66, 72].

During the generation of CAFs, various factors in the TME induce CAF activation by stimulating certain distinct signaling pathways (Table 1). In addition to the regulatory molecules described above, inflammatory mediators such as IL-1β and IL-6 act through the nuclear factor-kappa B (NF-κB) and Janus kinase (JAK)-signal transducer and activator of transcription 3 (STAT3) signaling pathways, respectively, to promote the malignant progression of CAFs [29, 73]. Analogous to the classical activating mechanisms reported for normal fibroblasts, such as wound stimulation, CAFs respond to damage-associated molecular patterns (DAMPs) released by necrotic cancer cells, which subsequently induce the activation of the internal NOD-like receptor protein 3 (NLRP3) inflammasome signaling pathway and ultimately contribute to tumor growth and metastasis through the secretion of inflammasomes [74]. Furthermore, tumor-derived exosomes that contain different transmitters, such as the CD44v6/C1QBP complex, have exhibited significant facilitation of the activation of HSCs and thus direct CAFs to induce tumor metastasis as well as ECM remodeling [45]. Moreover, heat shock factor 1 (HSF1, a master regulator of the heat shock response) was also reported to primarily orchestrate concomitant stimulation of both the β-catenin and YAP/TAZ signaling pathways through Dickkopf-3 (DKK3, an HSF1 effector), consequently resulting in aggressive behaviors of CAFs [75, 76]. Of note, epigenetic changes are capable of initiating and maintaining a proinvasive phenotype of CAFs [77]. Albrengues et al. [77, 78] revealed that leukemia inhibitory factor (LIF) can induce a series of internal epigenetic modifications in fibroblasts, including alterations in STAT3 acetylation, phosphatase methylation of SH2-containing protein tyrosine phosphatase-1 (SHP-1) and JAK1 phosphorylation, ultimately stimulating the JAK1/STAT3 signaling pathway, which sustains the proinvasive activities of CAFs. Finally, CAF activation also depends on environmental stressors (ROS, matrix stiffening, etc.) [79–84] and DNA damage during radiation therapy [85].

**Table 1:** Various stimulating factors and their related activating mechanisms during cancer-associated fibroblast progression

Stimulating factors	Types	Activating mechanisms	Biology effects	Cancer models	Refs
TGF-β1	Growth factor	TGF-β1-SMAD signaling pathway	Induces tumor cell proinvasive properties	Colon cancer, Breast cancer	[35, 38]
SDF-1 (CXCL12)	Chemokine	SDF-1-CXCR4 autocrine signaling pathway	Maintains myofibroblast activation	Breast cancer	[38]
IL-1β	Proinflammatory cytokine	NF-κB signaling pathway	Enhances tumor-promoting inflammatory response	Squamous skin carcinoma	[29]
IL-6	Proinflammatory cytokine	JAK-ROCK-STAT3 signaling pathway	Facilitates CAF-induced ECM remodeling	Melanoma	[73]
DAMPs	Cell-necrosis-associated product	NLRP3 inflammasome signaling pathway	Promotes tumor growth and metastasis	Breast cancer	[74]
CD44v6/C1QBP complex	PDAC-derived exosomes	IGF-1 signaling pathway	Induces HSC activation, ECM remodeling and liver metastasis	PDAC	[45]
HSF-1	Transcription factor	Wnt and YAP/TAZ signaling pathway	Facilitates CAF aggressive behaviors (mediating ECM remodeling, cancer cell growth and invasion)	Breast, colorectal and ovarian cancer	[76]
LIF	Multifunctional cytokine	Epigenetic switch	Initiates and maintains CAF proinvasive phenotypes, promotes tumor invasion and ECM remodeling	Head and neck, lung and breast cancer	[77, 78]
ROS	Oxidative stress molecule	Autophagy and caveolin-1 dependent pathway	Promotes tumor migration, invasion and ECM remodeling	Breast cancer	[40, 79]
Matrix stiffening	Environmental stressor	SFK-YAP signaling pathway	Establishes a feed-forward self-reinforcing loop to maintain CAF phenotypes and enhance matrix remodeling	Breast cancer	[81]

Various stimulating factors of cancer-associated fibroblast activation and related activating mechanisms during their progression

*TGF-β1* transforming growth factor-beta 1, *SMAD* Drosophila mothers against decapentaplegic protein, *SDF-1* stromal-derived factor-1, *CXCL12* C-X-C chemokine ligand 12, *CXCR4* C-X-C chemokine receptor 4, *IL-1β* interleukin-1 beta, *NF-κB* nuclear factor-kappaB, *IL-6* interleukin-6, *JAK* Janus kinase, *ROCK* Rho-associated kinase, *STAT3* signal transducer and activator of transcription 3, *CAF* cancer-associated fibroblast, *ECM* extracellular matrix, *DAMPs* damage-associated molecular patterns, *NLRP3* NOD-like receptor protein 3, *PDAC* pancreatic ductal adenocarcinoma, *IGF-1* insulin-like growth factor-1, *HSC* hepatic stellate cell, *HSF-1* heat shock factor-1, *Wnt* Wingless/dint-1 protein, *YAP* yes-associated protein, *TAZ* Tafazzin, *LIF* leukemia inhibitory factor, *ROS* reactive oxygen species, *SFK* SRC-family protein tyrosine kinase

Although researchers have recently employed advanced technology, such as genetic lineage tracing [86], the origins of CAFs among most cancer types remain elusive due to the lack of exclusive markers for normal fibroblasts and CAFs [87]. Lineage tracing methods combined with single-cell spatial analyses are urgently needed to identify the exact contribution of each cell type and illustrate the detailed mechanism of CAF activation during cancer development.

## Heterogeneity and plasticity of CAFs

Due to the existence of multiple types of cellular precursors, the CAF population behaves as complex cells with various fibroblast phenotypes and distinct functions among many cancer types [88] (Table 2). During the past several years, several subtypes of CAFs in pancreatic ductal adenocarcinoma (PDAC) have been identified through the application of transcriptome analyses, but none of these subtypes was given a specific definition [89, 90]. Ӧhlund and colleagues [91] first discovered and identified two spatially divided and totally opposite subsets of CAFs—myofibroblastic CAFs (myCAFs) and inflammatory CAFs (iCAFs). myCAFs are located in direct proximity to cancer cells and have high α-SMA expression; iCAFs are located far more distantly from neoplastic cells and express less α-SMA but secrete more IL-6 and other inflammatory factors (e.g., IL-8, IL-11 and LIF), and they might participate in immune suppression and tumor cachexia by stimulating the STAT3 signaling pathway. Subsequently, the presence of myCAFs and iCAFs in pancreatic cancer was confirmed through droplet-based single-cell transcriptomics technology [92]. Notably, the researchers also termed a newly discovered CAF subpopulation “antigen-presenting CAFs” (apCAFs), and these cells express MHC class II and CD74 instead of classical costimulatory molecules. Coincidentally, a subpopulation observed previously that was able to present antigens and contribute to the suppression of T cell-mediated antitumor responses was analogous to apCAFs [93]. Another study in PDAC reported similar subtypes of the CAF population described above. According to the results of single-cell RNA sequencing, fibroblast population 1 (FB1) and fibroblast population 3 (FB3) [94] might represent the previously described iCAF and myCAF populations, respectively. Interestingly, in that study, the researchers found that FB3 also processed and presented antigens by expressing numerous MHC-II-associated genes, indicating that FB3 might be a mixed population comprising myCAFs and apCAFs. Furthermore, a recent study further assessed the intertumor and intratumor heterogeneity of human PDAC-derived CAFs [95]. At least four subtypes of CAFs were characterized by different mRNA expression profiles, and periostin (POSTN), myosin-11 (MYH11), and podoplanin (PDPN) were selected as biomarkers for subtype A to C CAFs. Moreover, subtype A CAFs, which are located in the tumor invasive front, are associated with tumor capsule formation and metastatic progression. The subtype B population might be related to a poor prognosis, while subtype C CAFs appear to be related to a favorable clinical prognosis of patients with cancer. Various CAF subpopulations have been reported in human breast cancer. For example, four different CAF subsets (S1-S4) are classified based on their diverse expression of fibroblast markers (e.g., CD29, FAP, α-SMA, PDGFRβ, FSP1 and caveolin 1 (CAV1)) [96]. Both the CAF-S1 and CAF-S4 subsets exhibit protumorigenic properties, while the CAF-S1 subset enhances the differentiation, recruitment and activation of Treg cells, thereby facilitating immune suppression of tumors; the properties of this CAF-S1 subset are similar to those of the CAF-S1 subset observed in ovarian cancer [97]. Another study on axillary lymph nodes [98] further indicated that the CAF-S1 subset promotes cancer cell migration and EMT initiation mainly by secreting CXCL12 and TGF-β, while the CAF-S4 subset facilitates the migration and invasion of tumor cells through the NOTCH pathway. Additionally, the presence of myCAFs, iCAFs and apCAFs in breast cancer was recently documented, and these cells were previously identified in PDAC [99]. In addition, Bartoschek et al. [100] defined another four subpopulations of CAFs—vCAFs, mCAFs, cCAFs and dCAFs—according to their distinct cellular sources using single-cell RNA sequencing. vCAFs, mCAFs, and dCAFs appear to originate from perivascular cells, resident fibroblasts, and malignant cells, respectively. In addition, CAF subpopulations with different fibroblast phenotypes have also been detected in oral squamous cell carcinoma (OSCC) [101], colorectal cancer [102] and mesenchymal high-grade serous ovarian cancer (HGSOC) [97].

**Table 2:** Phenotypic and functional heterogeneity of cancer-associated fibroblasts exhibited in distinct tumor types

Cancer types	Subtypes	Characteristic markers/ expression/secretion	Functions	Refs
PDAC	myCAFs (pCAFs)	α-SMA, TAGLN, MYL9, TPM1, TPM2, POSTN and MMP11	Tumor proliferation, Migration, Invasion and ECM remodeling	[92, 93, 103]
	iCAFs (pCAFs)	PDGFRα, HAS1, HAS2, IL-6, IL-8, IL-11, CXCL1, CXCL2 and CCL2	Immune suppression, Cachexia and Chemoresistance	[92, 93, 103]
	apCAFs (pCAFs)	MHC class II, H2-Aa, H2-Ab1 and CD74	Antigen-present, Immune modulation	[93, 94, 103]
PDAC	CAF-A	POSTN	Tumor proliferation, Invasion, Metastasis	[95]
	CAF-B	MYH11	Lymph-node metastasis, Prognostic factor (adverse)	[95]
	CAF-C	PDPN	Immune promotion, Prognostic factor (favorable)	[95]
Breast cancer	CAF-S1	CD29, FAP, α-SMA, PDGFRβ, FSP1 and CXCL12	Tumor proliferation, Migration, Lymph-nodes metastasis, Immune suppression and EMT initiation	[96, 98]
	CAF-S2	Not reported	Not reported	[96, 98]
	CAF-S3	CD29, FSP1, PDGFRβ	Not reported	[96, 98]
	CAF-S4	CD29, FSP1, PDGFRβ and α-SMA	Tumor invasion, Migration, Lymph-nodes metastasis	[96, 98]
Breast cancer	myCAFs	α-SMA, ACTA2, TAGLN, MYL9, IGFBP-3 and TNC	Tumor proliferation, Migration, Invasion, Angiogenesis and EMT	[99]
	iCAFs	Ly6c1, CLEC3B, HAS1, DPT and COL14A1	Tumor proliferation, Metastasis, Angiogenesis, Immune evasion and Chemoresistance	[99]
	apCAFs	CD74, H2-Aa, H2-Ab1, H2-Eb1, KRT18 and FSP1	Antigen-present, Immune modulation	[99]
Breast cancer	vCAFs/cCAF (proliferative segment of vCAFs)	Notch3, EPAS1, COL18A1 and NR2F2 (perivascular cells)	Angiogenesis	[100]
	mCAFs	Fibulin-1, PDGFRα and CXCL14 (resident fibroblasts)	Immune regulation	[100]
	dCAFs	SCRG1 (malignant cells)	Not reported	[100]
Breast cancer	CD10^+^GPR77^+^	CD10 and GPR77	Chemoresistance	[104]
OSCC	CAF-N	HA, MMPs	Tumor invasion, Immunosuppression	[101]
	CAF-D	TGF-β	Tumor migration	[101]
Colorectal cancer	CAF-A	MMP2, DCN, αFAP and COL1A2	ECM remodeling	[102]
	CAF-B	α-SMA, ACTA2, TAGLN and PDGFA	Not reported	[102]
HGSOC	CAF-S1	CD29, FAP, αSMA, FSP1, PDGFRβ and CXCL12β	Tumor proliferation, Immune suppression	[97]
	CAF-S2 (non-activated)	Not reported	Not reported	[97]
	CAF-S3 (non-activated)	CD29, FSP1 and PDGFRβ	Not reported	[97]
	CAF-S4	CD29, αSMA, FSP1 and PDGFRβ	Tumor proliferation	[97]
PDAC/Oral/Colon/Bladder/Intestinal cancers	rCAFs	Meflin, BMP-4, Hedgehog and IKKβ	Antitumoral effect	[10, 105–111]

Multiple phenotype and function heterogeneous cancer-associated fibroblast subsets in distinct tumor types

*PDAC* pancreatic ductal adenocarcinoma, *myCAFs* myofibroblastic cancer-associated fibroblasts, *iCAFs* inflammatory cancer-associated fibroblasts, *apCAFs* antigen-presenting cancer-associated fibroblasts, *pCAFs* cancer-promoting cancer-associated fibroblasts, *α-SMA* alpha smooth muscle actin, *TAGLN* transgelin, *MYL9* myosin light chain 9, *TPM1* tropomyosin 1, *TPM2* tropomyosin 2, *POSTN* periostin, *MMP11* matrix metalloproteinase 11, *PDGFRα* platelet-derived growth factor receptor alpha, *HAS1* hyaluronan synthase 1, *HAS2* hyaluronan synthase 2, *IL-6* interleukin-6, *IL-8* interleukin-8, *IL-11* interleukin-11, *CXCL1* C-X-C chemokine ligand 1, *CXCL2* C-X-C chemokine ligand 2, *CCL2* C–C chemokine ligand 2, *MHC class II* major histocompatibility complex class II, *H2-Aa* histocompatibility 2 class II antigen A alpha, *H2-Ab1* histocompatibility 2, class II antigen A, beta 1, *CD74* cluster of differentiation 74, *ECM* extracellular matrix, *MYH11* myosin-11, *PDPN* podoplanin, *CD29* cluster of differentiation 29, *FAP* fibroblast activation protein, *PDGFRβ* platelet-derived growth factor receptor β, *FSP1* fibroblast-specific protein 1, *CXCL12* C-X-C chemokine ligand 12, *EMT* epithelial-mesenchymal transition, *ACTA2* actin alpha 2, *IGFBP-3* IGF-binding protein 3, *TNC* Tenascin-C, *Ly6c1* lymphocyte antigen 6 complex, locus C1, *CLEC3B* C-type lectin domain family 3, member B, *DPT* dermatopontin, *COL14A1* collagen type XIV alpha 1, *H2-Eb1* histocompatibility 2, class II antigen E, beta 1, *KRT18* keratin 18, *EPAS1* endothelial PAS domain protein 1, *COL18A1* collagen, type XVIII, alpha 1, *NR2F2* nuclear receptor subfamily 2 group F member 2, *CXCL14* C-X-C chemokine ligand 14, *SCRG1* scrapie responsive gene 1, *CD10* cluster of differentiation 10, *GPR77* G protein-coupled receptor 77, *OSCC* oral squamous cell carcinoma, *HA* hyaluronan, *TGF-β* transforming growth factor beta, *MMP2* matrix metalloproteinase 2, *DCN* decorin, *COL1A2* collagen type 1 Alpha 2, *PDGFA* platelet derived growth factor A, *HGSOC* high-grade serous ovarian cancer, *rCAFs* cancer-restraining cancer-associated fibroblasts, *BMP-4* bone morphogenetic protein 4, *IKKβ* inhibitor kappa B kinase beta

CAFs are composed of not only heterogeneous subsets with distinct phenotypes but also heterogeneous subpopulations with diverse functions [9, 30]. Observations indicate that two functionally different populations of CAFs, cancer-promoting CAFs (pCAFs) and rCAFs, may exist [112]. Generally, most CAF subsets function as pCAFs rather than rCAFs. Studies have revealed that pCAFs mainly express FAP-α or α-SMA to suppress antitumor immunity through multiple pathways [38, 93, 96, 103, 113]. Modulators secreted by pCAFs, such as TGF-β, IL-6 and CXCL12, are able to promote the proliferation and invasion of cancer cells [114]. However, a recent study indicated that one of the CAF subsets in PDAC that expresses meflin (one potential marker) exerts antitumor effects on both mouse models and human cells, and this subset was subsequently identified as rCAFs [10]. Importantly, the presence of rCAFs is not limited to the context of PDAC [105–107]. Patel et al. [108] reported a myofibroblastic CAF subpopulation that inhibited cancer cell stemness by secreting bone morphogenetic protein 4 (BMP-4) in oral carcinoma. In other tumor types, including colon [109], bladder [110] and intestinal cancers [111], tumor-suppressive roles of CAFs have also been reported, suggesting a wide distribution of rCAFs across various types of cancer. However, considering the lack of in-depth phenotypic and functional characterization of CAFs, further explorations of CAF heterogeneity in most other cancer types are currently extremely difficult.

As CAFs contain multiple heterogeneous subpopulations, researchers have recently debated whether these diverse subtypes are able to interconvert, which would indirectly confirm the plasticity of CAFs. Several studies indicate that the answer to these questions is “yes”. For example, iCAFs in pancreatic cancer have been reported to be able to transform into myCAFs upon the activation of TGF-β signaling or the inhibition of the IL-1-induced JAK/STAT signaling pathway, suggesting potential plasticity between these two cellular subtypes [99]. Furthermore, research has also discovered an intermediate state between the iCAF and myCAF phenotypes termed α-SMA + p-STAT3 + cells, which might subsequently be a potential target for tumor immunotherapies [99]. Moreover, in colorectal cancer, CAF-A cells (one of the CAF subtypes) were reported to be capable of converting into CAF-B cells (another CAF subtype) during the transformation of normal fibroblasts into CAFs [102]. In addition to the research described above, few studies have recently reported CAF plasticity. Currently, in-depth research on many other reported important pathways, such as the epidermal growth factor receptor (EGFR), Wnt and Hippo signaling pathways [115], and improved recognition of the epigenetic regulation of CAF states are required to help improve our understanding of CAF plasticity [87].

## Interaction between CAFs and the immune microenvironment in tumors

**Fig. 2:**
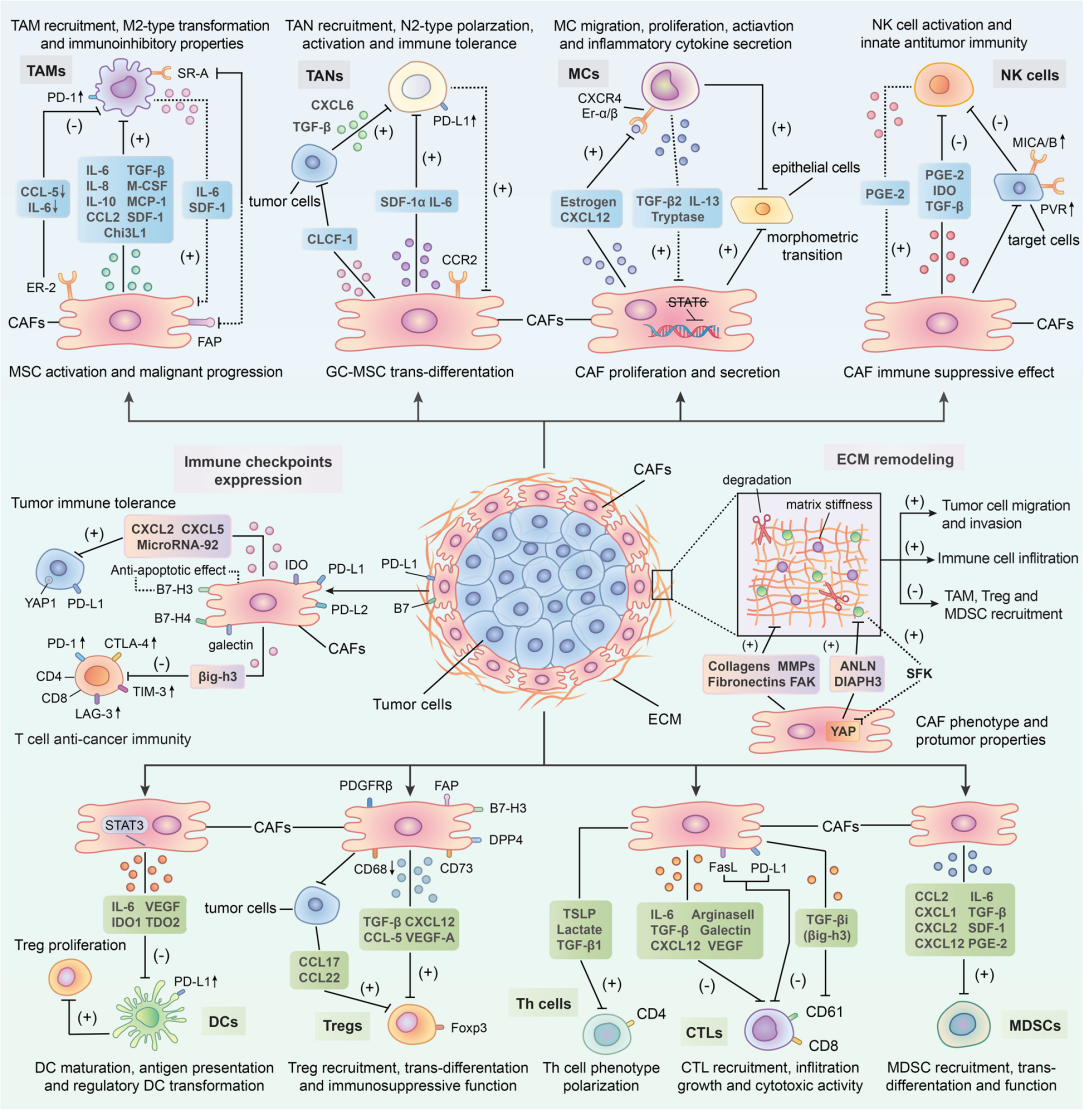
Crosstalk between cancer-associated fibroblasts (CAFs) and immune components in the tumor immune microenvironment (TIME). CAFs can orchestrate an immunosuppressive TME via interacting with the immune microenvironment in tumor. Through the secretion of multiple chemokines, cytokines and other effector molecules such as transforming growth factor-beta (TGF-β), interleukin-6 (IL-6), C-X-C chemokine ligand 12 (CXCL12), C–C chemokine ligand 2 (CCL2), stromal-derived factor-1 (SDF-1), vascular endothelial growth factor (VEGF) along with indoleamine 2,3-dioxygenase (IDO) and prostaglandin E2 (PGE2), CAFs modulate immune cells-mediated antitumor immunity through the following pathways: Promoting the trans-differentiation or polarization of immune cells such as tumor-associated macrophages (TAMs), tumor-associated neutrophils (TANs), mast cells (MCs), dendritic cells (DCs) and T lymphocytes into certain protumorigenic cell subsets; Facilitating the activities of immune inhibitory cells in terms of recruitment, activation and immunosuppressive effects including M2-type TAMs, N2-type TANs, regulatory DCs (rDCs), regulatory T(Treg) cells and myeloid-derived suppressor cells (MDSCs); Restricting the cytotoxic activity and cytokines production of effector immune cells like natural killer (NK) cells and cytotoxic T lymphocytes (CTLs). Notably, several infiltrating immune cells such as TAMs, TANs, MCs and DCs can in turn exert promoting effect on CAFs activation and function, thereby contributing to the formation of immune suppressive loops. Moreover, CAFs can also upregulate the expression of immune checkpoint molecules such as programmed cell death protein 1 (PD-1)/programmed death receptor ligand-1 (PD-L1) and cytotoxic T lymphocyte associate protein-4 (CTLA4)/B7 in both themselves and other cells in the TME to induce T-cells dysfunction. Meanwhile, CAFs are able to remodel extracellular matrix (ECM) to facilitate immune suppression through the production of fibronectin, collagen and metalloproteinases (MMPs) as well as the activation focal adhesion kinase (FAK) signaling pathway. Finally, immune checkpoint molecule overexpression on CAF surface as well as matrix deposition around would inhibit CAF apoptosis and facilitate their activation and function

Based on accumulating evidence, CAFs in the TME play important roles in regulating the antitumor activities of tumor-infiltrating immune cells, including innate and adaptive immune cells, in the TIME [7, 116]. In addition, they promote the expression of immune checkpoint molecules and ECM remodeling to indirectly influence the recruitment and activity of immune cells [116]. Through the secretion of cytokines, chemokines and other effector molecules, including TGF-β, CXCL2, collagens, MMPs and laminin, CAFs can prompt immune cells to participate in the occurrence and development of cancer, along with facilitating the degradation and remodeling of the ECM [117, 118]. Of course, some noteworthy effects of several immune cells on CAFs have also been identified [29, 119]. To date, many studies have shown that interactions between CAFs and immune cells as well as other immune components are capable of modulating the TIME and thus inhibiting the antitumor immune response (Fig. 2) [120, 121].

### Interaction between CAFs and innate immune cells in the TME

#### Interaction between CAFs and tumor-associated macrophages (TAMs)

Macrophages that infiltrate tumors, known as tumor-associated macrophages (TAMs), are classified into two distinct subsets that are activated by different polarizing cytokines, termed M1 (lipopolysaccharide (LPS) alone or with Th1 cytokines) and M2 (Th2 cytokines) [122]. M1-type macrophages mainly behave as an antitumor role in the TIME by mediating antibody-dependent cellular cytotoxicity and producing ROS and tumor necrosis factor (TNF) [123], whereas M2-type macrophages exhibit tumor-promoting activity by contributing to the activation of tumor angiogenesis, immune suppression, invasion and metastasis of cancer cells and remodeling of the ECM [124, 125].

As a key component of the TIME, TAMs play critical roles in its modulation, especially in tumor immune suppression [126, 127]. TAMs are the most prominent immune cells in the vicinity of CAF-populated areas, suggesting tight interactions between these two cell types [128]. High expression of both CAF and TAM markers, such as α-SMA, FAP and S100 calcium binding protein A4 (S100A4), along with CD163 and CD209, is related to worse clinical prognosis of patients with some tumors [128, 129]. A substantial number of studies have shown that CAFs promote the recruitment of monocytes (macrophage precursors) and their differentiation into protumorigenic macrophage subsets (M2-type TAMs) via multiple regulatory molecules, thereby impairing responses from effector T cells and inducing immune suppression in the TME [130]. For example, in breast cancer, by secreting monocyte chemotactic protein-1 (MCP-1), SDF-1 and chitinase 3-like 1 (Chi3L1), CAFs are able to facilitate monocyte migration and enhance the tendency of these cells to polarize into the M2 phenotype [131, 132]. Furthermore, a similar effect of CAFs on TAMs was discovered in prostate carcinoma [133, 134]. Moreover, Mace et al. [135] documented the central role of CAF-derived macrophage colony-stimulating factor 1 (M-CSF1), IL-6, and CCL2 in monocyte recruitment and the increased M2/M1 macrophage ratio in pancreatic cancer. Other cytokines, including IL-8, IL-10, TGF-β and CCL2 (in skin tumors), secreted by CAFs have also been demonstrated to promote the recruitment of monocytes and their transformation into M2 macrophages [136–138]. In addition to facilitating macrophage recruitment and trans-differentiation, more importantly, CAFs are capable of inducing the immunoinhibitory properties of TAMs. Utilizing flow cytometry analysis, Gordon et al. [139] observed increased expression of programmed cell death protein 1 (PD-1) on the cell surface of CAF-induced M2-type TAMs. A high level of PD-1 expression in TAMs was proven to be involved in both innate and adaptive antitumor immune response suppression by subsequent studies, including decreasing their own phagocytic potency against tumor cells and inhibiting T-cell infiltration and proliferation [140]. In contrast to their stimulatory effect on TAMs, CAFs might also inhibit some aspects of TAM activities. Estrogen receptor alpha (ERα), for instance, whose expression on CAFs suppresses macrophage infiltration and restricts prostate cancer invasion, is mediated by decreased CCL5 and IL-6 expression. Mechanistically, Mazur et al. [141] revealed the importance of FAP (a CAF marker) in the communication between CAFs and TAMs. The authors found that FAP participated in the interaction between CAFs and SR-A (class A scavenger receptors) + TAMs mainly by cleaving type I collagen and increasing macrophage adhesion.

Reciprocally, TAMs with the M2 phenotype regulate CAF activation and progression as well [119, 142]. In the study by Comito et al. [133], aside from confirming the promoting effect of CAFs on TAMs, M2-type macrophages were also able to enhance EMT progression to stimulate CAF activation by secreting soluble factors such as IL-6 and SDF-1. Moreover, TAMs were recently shown to influence the trans-differentiation and activity of MSCs, one of the cellular precursors of CAFs [48]. For instance, Zhang et al. [143] observed that macrophages could facilitate MSCs to acquire CAF-like properties and a proinflammatory phenotype to remodel the inflammatory microenvironment, which potentiated the oncogenic transformation of gastric epithelial cells. Additionally, in an in vitro coculture study, TAM-like macrophages were reported to induce both the proliferation and invasion of CAF-like BM-MSCs, thereby contributing to the progression of neuroblastoma [48]. Subsequently, activated CAFs induced by macrophages further enhance TAM activity, and consequently make up a positive loop that promotes cancer development and immune inhibition in the TME.

Recently, studies regarding the effect of CAFs on TAMs have been continuously reported, whereas the effect of macrophages on CAFs has not been comprehensively investigated and clarified. Further investigations of the mechanisms underlying CAF-TAM interactions in the TME are needed to advance current cancer-targeted therapies.

#### Interaction between CAFs and tumor-associated neutrophils (TANs)

Increasing evidence indicates that tumor-associated neutrophils (TANs), a significant component of the TIME, also exhibit phenotypic heterogeneity and functional versatility [144, 145]. Analogous to the dichotomy of TAMs (M1 and M2), neutrophils can acquire an antitumor phenotype (N1) or a protumorigenic phenotype (N2) based on whether they are activated by TGF-β [146–148]. But unlike TAMs, the difference between N1 and N2 TAN phenotypes relies on the distinct degree of activation rather than different polarizing molecules [149].

Notably, CAFs might be able to modulate the polarization of TANs. As a recent study of hepatocellular carcinoma reported, CAF-derived cardiotrophin-like cytokine factor 1 (CLCF1) induces the polarization of N2-phenotype neutrophils by upregulating CXCL6 and TGF-β expression in tumor cells, thereby facilitating tumor progression [150]. More importantly, CAFs probably participate in all stages of the malignant progression of TANs and ultimately suppress the antitumor immune response in the TME. Through the secretion of SDF-1α, CAFs are able to recruit peripheral neutrophils to tumors [151]. Moreover, C-X-C chemokine receptor 2 (CXCR2), a cytokine receptor that is expressed by CAFs, was proven to be a primary factor participating in the recruitment of neutrophils in tumors, indicating that CAFs might enhance the migration of TANs in a CXCR2-dependent manner [152, 153]. Next, CAF-derived IL-6 stimulates the STAT3 signaling pathway in TANs, consequently inhibiting the activity of T cells and inducing immune tolerance through the expression of PD-1/programmed death ligand 1 (PD-L1) [151]. In addition, Zhu et al. [154] discovered a bidirectional interaction between gastric cancer mesenchymal stem cells (GC-MSCs) and neutrophils. On the one hand, GC-MSCs can induce the chemotaxis and activation of neutrophils and sustain their survival through the IL-6-mediated STAT3-extracellular regulated protein kinases 1/2 (ERK1/2) axis. On the other hand, activated TANs, in turn, are capable of promoting the differentiation of MSCs into CAFs. Overall, the specific mechanisms underlying the mutual effects of CAFs and TANs on each other remain unclear due to the limited number of reports.

#### Interaction between CAFs and mast cells (MCs)

In recent decades, studies of mast cells (MCs) have placed more focus on their roles in cancer than on their roles in allergic diseases [155, 156]. As a component of the TIME, interestingly, MCs exert dual effects on tumor progression—both promotion and inhibition of tumor growth—which depend on the specific MC localization, cancer type and the degree of tumor progression [157–161]. As cancer promoters, on the one hand, MCs contribute to the stimulation of angiogenesis and lymphangiogenesis along with the degradation of ECM by producing different pro-angiogenic molecules (vascular endothelial growth factor (VEGF)-A, VEGF-B, FGF-2, heparin, histamine and stem cell factor (SCF)) [162–166], lymphangiogenic molecules (VEGF-C and VEGF-D) [167], matrix metalloproteinase-9 (MMP-9) and proteases (tryptase and chymase) [168, 169]. On the other hand, as antitumor effectors, MCs produce mediators (e.g., tryptase, chondroitin sulfate, TNF, IL-1 and IL-6) that increase antitumor inflammatory reactions, inducing tumor apoptosis and decreasing the invasiveness of cancer cells [170, 171].

Excess numbers of CAFs and MCs in tumor islets are strongly associated with the aggressiveness of cancer, and their interactions directly contribute to tumor progression [172, 173]. In prostate cancer, with the overexpression of estrogen inside, CAFs can potentiate MC proliferation, migration and inflammatory cytokine secretion and thus exhibit protumorigenic effects [174]. Meanwhile, CAF-derived CXCL12, induced by estrogen, was observed to be involved in the recruitment of MCs by combining with CXCR4 [174]. Furthermore, Ma et al. [175] discovered that PSCs could facilitate the activation and proliferation of MCs as well. This study also identified the stimulatory effect of MCs on CAFs. IL-13 and tryptase, which are released by MCs, conversely stimulate CAF proliferation in a TGF-β2-STAT6-independent manner [175]. Increasing CAFs subsequently resulted in the formation of a fibrotic TME and ultimately suppressed antitumor immunity and therapeutic responses [175]. Moreover, MCs in neurofibroma have also been reported to be capable of promoting CAF activity, such as enhancing the proliferation and secretion of CAFs through the TGF-β signaling pathway, thereby increasing the protumor effects of CAFs [173]. Additionally, a recent study in a microtissue model of prostate cancer revealed cooperation between MCs and CAFs, which induced the early malignant morphological transition of benign epithelial cells [176]. To date, research on the correlation between MCs and CAFs in tumors is still lacking. Considering the unique role of MCs and their mediators in the TIME, studies elucidating how CAF-MC interactions are implicated in tumor immunity are required to provide better immunotherapy and clinical services.

#### Interaction between CAFs and natural killer (NK) cells

Natural killer (NK) cells, members of the innate immune system, naturally respond to tumor cells [177–180]. The activity of NK cells depends on the expression and stimulation of activating or inhibitory receptors on the cell surface [181]. NK cell-activating receptors include NK group 2D (NKG2D), NKp30, NKp44, NKp46 and DNAX accessory molecule 1 (DNAM-1), while killer immunoglobulin-like receptors (KIRs) and CD94/NK group 2A (NKG2A) expressed on NK cells are inhibitory receptors [179, 182]. In solid tumors, various soluble inhibitory factors and cell types, such as CAFs, comprise the immunosuppressive TME, contributing to the impaired functionality of infiltrating NK cells [183, 184].

An increasing number of studies indicate that CAFs exert inhibitory effects on NK cells through multiple processes, including NK receptor activation, cytotoxic activity and cytokine production, in a direct or indirect manner [9, 185]. For example, under the influence of melanoma-associated fibroblasts, both the expression of NKp30, NKp44 and DNAM-1 activating receptors on the cell surface and the formation of cytolytic granules in NK cells are suppressed, which mainly depends on the prostaglandin E2 (PGE2) released by CAFs [186]. In hepatocellular carcinoma, CAFs educate NK cells to transition into an inactivated phenotype through PGE2 and indoleamine 2,3-dioxygenase (IDO) and create an unresponsive state in antitumor immunity [187]. Interestingly, NK cells themselves can facilitate the formation of the suppressive loop induced by CAFs via promoting the secretion of PGE2 [188]. Certainly, CAFs can also restrict the activity and function of NK cells indirectly by modulating the expression of their activating receptor-associated ligands on tumor cells. For instance, according to Ziani et al. [189], CAFs in melanoma reduce the expression of MICA/B (two ligands of NK-activating receptors) on tumor cells, thereby suppressing NKG2D-dependent cytotoxic activity and IFN-γ secretion. Another study reported that a reduction in poliovirus receptor (PVR, a ligand of an NK-activating receptor) expression on the cell surface plays a critical role in the CAF-mediated suppression of NK cell killing activities [190]. In addition, macrophages induced by CAFs are reported to inhibit NK cell cytotoxicity and activation, which indicates that CAFs can regulate NK cells through other immune cells [137]. When cocultured with NK cells, higher PGE2 expression is detected on CAFs than on normal fibroblasts [188], suggesting that NK cells can influence the certain protein expression in CAFs as well. However, currently, only few studies have assessed the effect of NK cells on CAFs, and further investigations are needed to clarify this interacting progress.

The detailed mechanism of crosstalk between CAFs and NK cells is complicated and multiple effector molecules might participate in the interaction. TGF-β has been widely reported to be a key cytokine connecting CAFs with NK cells in tumors [191]. Substantial studies have proven that CAF-secreted TGF-β significantly inhibits the activation and cytotoxic activity of NK cells [192]. One of the possible mechanisms is that TGF-β reduces the production of interferon-γ (IFN-γ) and downregulates cell surface activating receptors, such as NKG2D [193, 194]. For instance, TGF-β can inhibit DNAX-activation protein 12 (DAP12) transcription and reduce the expression of NKp30 and NKG2D by stimulating miR-183, thus silencing NK cells [195]. Moreover, Viel et al. [196] reported that TGF-β1 selectively downregulated NKp30, NKp46, NKG2D and DNAM-1 expression in vitro through the activation of the SMAD2/3-dependent signaling pathway. In addition to TGF-β, the exploration of other related molecules is still ongoing.

#### Interaction between CAFs and dendritic cells (DCs)

Tumor-infiltrating dendritic cells (DCs), a heterogeneous group consisting of diverse subpopulations, play a crucial role in the activation and regulation of innate and adaptive immune responses in the TIME through the high expression of class I and class II MHC complexes, adhesion molecules and costimulatory molecules [197, 198]. In recent years, several investigations have illustrated that CAFs can drive immune evasion of tumor cells by blocking DC maturation, antigen presentation and their associated adaptive immune responses. However, their in-depth mechanisms remain unclear. By activating the IL-6-mediated STAT3 pathway, CAFs in hepatocellular carcinoma can recruit normal DCs and induce them to transdifferentiate into regulatory DCs (rDCs), disabled DCs that express costimulatory molecules at a low level and hardly present antigens, but secrete inhibitory cytokines such as IDO [199]. Further studies have revealed the importance of regulatory DC-derived IDO in the promotion of T cell anergy and Treg cell proliferation, which consequently results in the restriction of T cell-mediated immunity [200]. Another study of lung cancer indicated that both CAF-released IDO1 and tryptophan 2,3-dioxygenase (TDO2) induced by lung cancer-derived galectin-1 are responsible for the impaired differentiation and function of DCs through the degradation of tryptophan [201, 202]. In addition, studies have demonstrated that VEGF produced by CAFs is involved in the abnormal differentiation and impaired antigen-presenting function of DCs via inhibiting the activation of NF-κB [203, 204]. Meanwhile, VEGF is also able to facilitate immune tolerance by upregulating PD-L1 expression on the DC surface [205].

### Interaction between CAFs and adaptive immune cells in the TME

#### Interaction between CAFs and T lymphocytes

T lymphocytes play a key role in modulating the adaptive immune response, and they comprise different subpopulations, such as Treg cells, helper T (Th) cells and cytotoxic T lymphocytes (CTLs) [206]. Numerous studies have illustrated the role of CAFs in modulating T cell activities and functions.

Treg cells with high Foxp3 expression are known to have crucial functions in the restriction of antitumor immunity [207]. Utilizing histochemical staining, Kinoshita et al. [27] confirmed that Treg cells are located adjacent to CAFs. Furthermore, the infiltration of both Foxp3 + Tregs and CAFs in the tumor stroma was correlated with a poor prognosis according to clinical data [27]. These results all indicate that potential crosstalk between CAFs and Treg cells might exist. Evidence of the interaction between CD70 + CAFs and naturally occurring Tregs has already been reported [208]. In a study of colorectal cancer, researchers revealed that CAFs stimulate the migratory activity of Treg cells and markedly increase their frequency in tumor sites [208]. Moreover, the recruitment of CD4 + CD25 + Treg cells to CAFs also depends on the chemokine CCL5 according to studies examining breast cancer [209, 210]. Other molecules, such as VEGF-A, one of the growth factors released by CAFs, have been observed to directly or indirectly participate in Treg cell induction and maintenance [211, 212]. In addition to promoting the recruitment and infiltration of Treg cells, CAFs also promote their transformation to ultimately induce immune suppression. As shown in the study by Chen et al. [213], CAF-derived TGF-β can facilitate the differentiation of naïve T cells into CD4 + CD25 + Treg cells by inducing the expression of the Foxp3 gene in T lymphocytes. Additionally, FAP + PDGFRβ + CAFs in breast cancer, also termed CAF-S1 cells (introduced earlier in the review), were proven to not only enhance the migration of CD4 + CD25 + T cells by releasing CXCL12 but also express CD73, dipeptidyl peptidase IV (DPP4) and B7H3, enabling them to transform CD4 + T cells into Foxp3 + Treg cells [96]. Recently, Zhao X and colleagues [214] discovered that downregulation of CD68 in CAFs facilitates the secretion of CCL17 and CCL22 from tumor cells and further indirectly increases the infiltration of Treg cells. However, interestingly, Özdemir et al. [105] obtained the opposite result from the experiment: the exhaustion of myofibroblasts in PDAC increases the proliferation of CD4 + Foxp3 + Tregs and subsequently inhibits immune surveillance, suggesting that a possible mixed and dual relationship might exist between CAFs and Treg cells.

Th cell subsets mainly include Th1, Th2, and Th17 cells, which are mostly differentiated from naïve CD4 + T cells [215]. By secreting various specific cytokines, Th1 and Th2 cells participate in cellular and humoral immunity, respectively [216]. Several reports have shown the great influence of CAF-associated activities on Th cell polarization, while their specific effects remain unclear. For example, when CAF activation proteins are targeted by a DNA vaccine, the polarization of the Th2 subset is suppressed at the same time, indicating that activated CAFs might promote the differentiation above [217]. Subsequently, De Monte et al. [218] found that thymic stromal lymphopoietin (TSLP) produced by activated CAFs in pancreatic cancer functions to promote Th2 polarization. In prostate cancer, in contrast, CAFs drive the polarization of naïve CD4 + T cells from the Th2 to Th1 phenotype by stimulating the miR21/Toll-like receptor 8 (TLR8) axis through the release of lactate [219]. In addition, by producing TGF-β1, CAFs can facilitate Th17 cell differentiation in vivo and disease development [220]. Altogether, CAFs modulate the transformation of most Th cells into immunoinhibitory subpopulations in tumors to create an immunosuppressive and cancer-adaptive TME and then exert a proinvasive effect on cancer cells.

CD8 + T cells, also called CTLs, mediate cytotoxic activities mainly by inducing the apoptosis of tumor cells, which is considered the most critical component of antitumor immunity [221, 222]. A substantial number of studies have reported the interactions between CAFs and CD8 + T cells and documented the inhibitory effect of CAFs on CD8 + T cell infiltration, growth and antitumor immunity [223]. Multiple factors account for the decreased infiltration of CD8 + T cells in the TME. For instance, by secreting cytokines such as CXCL12, activated PSCs are able to facilitate the trafficking of CD8 + T cells away from the juxta-tumoral compartment and thus reduce the frequency of infiltrating CTLs in tumor islets [25]. Subsequently, the importance of the CXCL12 signaling pathway in the regulation of tumor-infiltrating CD8 + T cell migration induced by FAP + CAFs, has been confirmed in several reports [224, 225]. Certainly, the physical barriers and hypoxia in the TME caused by CAF-mediated ECM modification are also responsible for T cell movement restriction [226]. CAFs release various angiogenic factors in response to hypoxia, such as VEGF, which leads to decreased cell adhesion molecule (e.g., intercellular cell adhesion molecule (ICAM)-1/2 and vascular cell adhesion molecule-1 (VCAM-1)) expression on endothelial cells [227]. Due to the lack of cell adhesion molecules, the extravasating progress of peripheral CD8 + T cells into tumor sites through the vasculature is hard to maintain [228]. In addition, CAFs can also reduce CD8 + T cell recruitment by releasing IL-6 and TGF-β, and inhibit their cytotoxic activities toward tumor cells as well [113, 229]. Further related clinical trials have indicated that IL-6 blockade therapy effectively improves the function of T cells and the prognosis of patients with cancer [113, 229]. According to the research of Goehrig et al. [230], CAFs can exert a direct suppressive effect on CD8 + T cell function, including their proliferation, activation and cytotoxic activity, through the secretion of βig-h3 (one ECM protein, also termed TGF-βi). Mechanistically, CAF-derived βig-h3 induces the combination of hydrogen peroxide inducible clone-5 (HIC-5) protein and Y505 phosphorylated Lck by binding to CD61 (one CD8 + T cell surface marker) and consequently decreases the transduction of T cell receptor (TCR) signaling [230]. Moreover, arginase II and galectin expressed in CAFs are also involved in the progression of suppressing CD8 + T cell proliferation and promoting T cell anergy [231–233]. Of note, as previously described, CAFs are capable of inhibiting CD8 + T cell cytotoxic function in indirect manners. CAFs not only blunt antigen presentation of DCs or NK cells by disturbing their normal differentiation [187, 199], but also induce immunoinhibitory subsets (e.g., TAMs and Treg cells) and immune checkpoint expression to impair effector T cell antitumor responses [130, 151]. Recently, in-depth research has revealed a possible suppressive mechanism by which CAFs in the TME might function in a similar manner to normal DCs, including participating in antigen sampling, processing and presentation and upregulating the expression of immune checkpoint molecules (factor associated suicide (FAS)/factor associated suicide ligand (FASL) and PD-1/programmed death ligand 2 (PD-L2)), thereby promoting a decrease in the number of CD8 + T cells and an increase in tumor cell viability [93]. Since CAFs can suppress the immune reaction in the TME by regulating the properties of various T cell subsets, targeted immunotherapies aimed at the CAF-T cell interaction might be effective at stimulating an impaired antitumor response.

In conclusion, CAFs facilitate the cancer-promoting phenotype transition of naïve T cells, enhancing immune inhibitory T lymphocyte function and suppressing the activity of effector T lymphocytes, thereby resulting in immune suppression in the TME. Currently, there is still a lack of studies reporting the effect of T lymphocytes on CAFs, which might be a novel potential direction for future research.

#### Interaction between CAFs and MDSCs

Originating from bone marrow, MDSCs are famous for their strong immunosuppressive activity in the TIME [234]. MDSCs mainly contain two cell subsets, termed polymorphonuclear MDSCs (PMN-MDSCs) and monocytic MDSCs (M-MDSCs), which are phenotypically and morphologically similar to neutrophils and monocytes, respectively [235, 236]. In contrast to MDSCs that are activated by bacteria and viruses, MDSCs in the TME exhibit less phagocytic activity while continuously releasing anti-inflammatory cytokines, ROS and nitric oxide (NO), thereby contributing to the promotion of cancer angiogenesis, invasion, metastasis and immune tolerance [237–239].

Recently, a novel MDSC subset, named circulating fibrocytes, was reported to exhibit phenotypic and functional similarity to CAFs, suggesting a possible association between MDSCs and CAFs [240]. By releasing various cytokines and chemokines, CAFs can facilitate the infiltration and generation of MDSCs and consequently suppress effector T cell antitumor activity. Evidence indicates the essential role of CCL2 in the recruitment of both PMN-MDSCs and M-MDSCs [28, 241]. As a major source of CCL2, CAFs might induce MDSCs to migrate into tumor sites by stimulating the STAT3 signaling pathway [225]. For example, CAFs in lung squamous cell carcinoma (LSCC) have been reported to promote peripheral C–C chemokine receptor (CCR)2 + monocyte migration via CCL2 and then reprogram them into M-MDSCs [242]. The accumulation of immunoinhibitory subpopulations (M-MDSCs) in the TME finally contributes to CD8 + T cell growth and IFN-γ production restriction [242]. Moreover, in hepatic carcinoma, Deng et al. [243] found that recruited monocytes can differentiate into M-MDSCs, and this transformation is induced by CAFs through IL-6 in a STAT3-dependent manner, which subsequently results in extensive inhibition of T cell proliferation and function. Another study described similar effects of CAF-secreted CXCL12 on monocytes in triple-negative (TN) breast cancers [244]. Recent research in esophageal squamous cell carcinoma confirmed the importance of CAF-secreted IL-6 in MDSC differentiation and observed that CAF-derived exosome-packed microRNA-21 (miR-21) is also responsible for the generation of M-MDSCs via activating STAT3 signaling [245]. In addition, under the inhibitory action of tranilast (a CAF suppressor), the expression of CAF-derived SDF-1, PGE2 and TGF-β1 is decreased, along with a low-level differentiation of original MDSCs [246]. These findings indicate that SDF-1, PGE2 and TGF-β1 probably participate in the differentiation and modulation of MDSCs [246]. Finally, CXCL1, a granulocytic chemokine produced by CAFs, might also be involved in PMN-MDSC recruitment [247].

### Interaction between CAFs and other immune cells

Certainly, other immune cells, such as monocytes and B cells, can crosstalk with CAFs as well. As we described above, CAFs are able to facilitate monocyte migration and trans-differentiation into M2-type TAMs [131, 132]. For B cells, only CXCL13 secreted by CAFs has been reported to enhance the recruitment of B cells [116]. Moreover, no other study has reported CAF-B cell interactions.

### Interaction between CAFs and other immune components in the TME

#### CAFs upregulate the expression of immune checkpoint molecules on the cell surface to induce immunologic tolerance

High expression of immune checkpoint molecules on the surface of T-cells and tumor cells has been identified as a main contributor to the dysfunction of T lymphocytes in the TME [248–251]. PD-L1 and PD-1, for example, are well-known checkpoint molecules. The binding of PD-L1 to its receptor PD-1 on activated T cells hampers antitumor immunity by counteracting T cell-activating signals [252].

CAFs themselves can express different ligands of immune checkpoint molecules on their cell surface, including PD-L1, PD-L2, B7-H3/H4, galectins and the enzyme IDO [93, 253–256]. Studies have demonstrated that the overexpression of PD-L1 and PD-L2 on CAFs among colon tumors, melanoma, carcinomas and lung cancer substantially induces T cell exhaustion and deactivation [93, 257–259]. Furthermore, α-SMA + CAFs expressing high levels of B7-H3 were recently shown to exhibit prolonged survival because of the antiapoptotic effect of this checkpoint molecule, and its presence also predicts a poor prognosis of gastric adenocarcinomas (GACs) [255, 260].

In addition to the upregulation of molecules on their own surface, CAFs also produce various types of cytokines and exosomes to upregulate checkpoint molecules on other cells, such as tumor cells and immune cells in the TME, which indirectly exert inhibitory effects on T cell function and antitumor responses. For instance, CAFs in pancreatic cancer have been reported to upregulate the expression of certain immune checkpoint molecules, including PD-1, cytotoxic lymphocyte-associated antigen-4 (CTLA-4), T cell immunoglobulin, mucin-domain containing-3 (TIM-3) and lymphocyte-activation gene-3 (LAG-3), on both CD4 + and CD8 + T cell surfaces, which consequently inhibits proliferating T cells and their specific recognition of tumor cells [261]. During the regulation of immune checkpoints, CAF-derived βig-h3 might play a crucial role in promoting the expression of certain immune checkpoint molecules [230]. When applying βig-h3-targeted depleting Ab therapy, researchers observed the reduced expression of PD-1 and TIM-3 on the tumor-specific CD8 + T cell surface along with the recovery of their proliferation and activity [230]. Moreover, IL-6 secreted by CAFs, as described before, can induce PD-L1 expression on neutrophils by activating the STAT3 signaling pathway to restrict effector T cell function [151]. Interestingly, CAF-derived factors involved in the upregulation of PD-L1 in different tumor cell types are distinct. Through the secretion of soluble factors like CXCL2, α-SMA + CAFs can increase PD-L1 expression in lung adenocarcinoma cells, thereby influencing antitumor immunity [262]. In melanoma and colorectal carcinoma, Li et al. [263] found that CAF-derived CXCL5 was involved in the expression of PD-L1 on the tumor cell surface in a PI3K/AKT-dependent manner. Recent studies have revealed some detailed intracellular signaling mechanisms. As shown in the research by Zhang et al. [264], CAFs in colorectal cancer facilitate extracellular signal regulated kinase 5 (ERK5) expression and phosphorylation to increase the synthesis of PD-L1 protein. Additionally, in human breast cancer, studies recently confirmed that microRNA-92 in CAF-derived exosomes targets LATS2 (a target gene of miR-92) and enhances the nuclear translocation of yes-associated protein 1 (YAP1); in this way, YAP1 binds to the enhancer region of PD-L1 to promote its transcriptional activity [265]. However, less is currently known about CAF induction of immune checkpoint molecule expression on other cells in the TME.

Overall, CAFs not only induce endogenous overexpression of checkpoint molecule ligands but also upregulate the expression of immune checkpoint molecules on other cells in the TME, thereby contributing to the impaired function of tumor-infiltrating T lymphocytes and immunologic tolerance. Certainly, further studies are needed to clarify the deeper mechanisms of CAF-induced immune checkpoint molecule expression, which might be a potential target for CAF-specific immunotherapies.

#### CAFs remodel the extracellular matrix (ECM) to facilitate immune suppression

The extracellular matrix (ECM) is a complex network consisting of different macromolecules, including collagens, fibrin, glycoproteins and proteoglycans, responsible for maintaining the architecture, integrity, development and homeostasis of normal tissue [18, 266, 267]. ECM alteration in the TME is a common phenomenon in tumor tissues and is usually related to cancer progression [268]. Many studies have demonstrated the pivotal role of CAFs in remodeling the ECM [17, 269]. By secreting multiple matrix proteins (e.g., fibronectin and type I collagen) and producing a variety of matrix metalloproteinases (MMPs), such as MMP-1 and MMP-3, CAFs can facilitate the degradation of normal ECM structure along with increasing matrix stiffness [270–274]. Moreover, CAFs also release the cytokine TGF-β1, a growth factor that is reported to be one of the most important regulators during ECM remodeling [275, 276]. The modified ECM, in turn, exerts promoting effects on CAF activation and protumorigenic function. A positive feedback loop between CAFs and the ECM has been identified by Calvo et al. [81]. Through activated YAP, CAFs are capable of upregulating the expression of several cytoskeletal regulators (e.g., anillin (ANLN) and diaphanous-related formin-3 (DIAPH3)) to contribute to ECM stiffening [81]. When the matrix becomes stiffer in the ECM, isometric tension within CAFs significantly increases and further facilitates YAP activation by stimulating Src family kinases (SFKs), consequently maintaining the CAF phenotype and their cancer-promoting properties [81].

Based on accumulating evidence, the modified ECM induced by CAFs is associated with the migration and invasion of cancer cells [17, 277, 278]. More importantly, this modified matrix participates in the induction of immune suppression within the TME. The CAF-remodeled ECM protein network serves as a physical barrier for immune cells, especially T lymphocytes, thus inhibiting their recruitment into cancer sites and subsequently reducing their opportunities to participate in the immune response in the TME [279, 280]. The collagen density of the ECM is able to determine the T cell distribution in the TME. Increased collagen deposition surrounding tumor cell clusters in lung tumors and pancreatic cancers was observed to restrict T lymphocyte access to contacting cancer cells [281, 282]. In addition, the accumulation of numerous matrix proteins in the ECM also results in a chronic hypoxia state in the TME^[[283]]^. As previously described, some soluble factors such as VEGF induced by hypoxia can decrease the effusion rate of circulatory T cells through tumor vessels and then reduce their infiltration [227, 228]. Further study revealed the critical role of focal adhesion kinases (FAKs, nonreceptor tyrosine kinases, including FAK1 and PYK2/FAK2), as fibrotic regulators, in the poor infiltration of CD8 + cytotoxic T cells induced by CAF-directed matrix deregulation [284]. The fibrous stroma of the ECM around tumor islets often blocks high-molecular-weight drugs and thus decreases the efficacy of cancer chemotherapy [285]. Recent studies have indicated that FAK-targeted inhibition can decrease the stromal density and consequently increase the responsiveness of tumors to chemotherapy and immunotherapy, suggesting that it might be a potential therapeutic target for tumor chemoresistance [286].

In addition, the CAF-modified ECM can modulate the activities of other immune cell populations as well. Abnormal cancerogenic collagenous matrix is involved in TAM recruitment and function [287]. For instance, the collagen-rich matrix induced by CAFs not only promotes monocyte migration and proliferation, but also shifts macrophage differentiation to M2 polarization (a protumorigenic cell subset) [288–291]. Reciprocally, TAM direct or indirect modulatory regulation of collagen deposition and geometrical organization gradually increase matrix rigidity and ultimately accelerate ECM remodeling progress [287]. Moreover, the ECM also facilitates the infiltration of other immunoinhibitory subpopulations. Increased collagen density or stiffness in the ECM leads to extensive FAK activation within cells, and activated FAKs subsequently drive the direct exhaustion of CD8 + T cells and enhance the recruitment of Tregs, MDSCs and TAMs, thereby contributing to the formation of an immunosuppressive TME [292, 293]. Altogether, the ECM has been demonstrated to crosstalk with several immune cells to induce immune suppression, whereas the effect of the ECM on other cell types, such as DCs and TANs, remains unclear.

## Therapeutic strategies for targeting CAFs to enhance the anticancer immune response

**Fig. 3:**
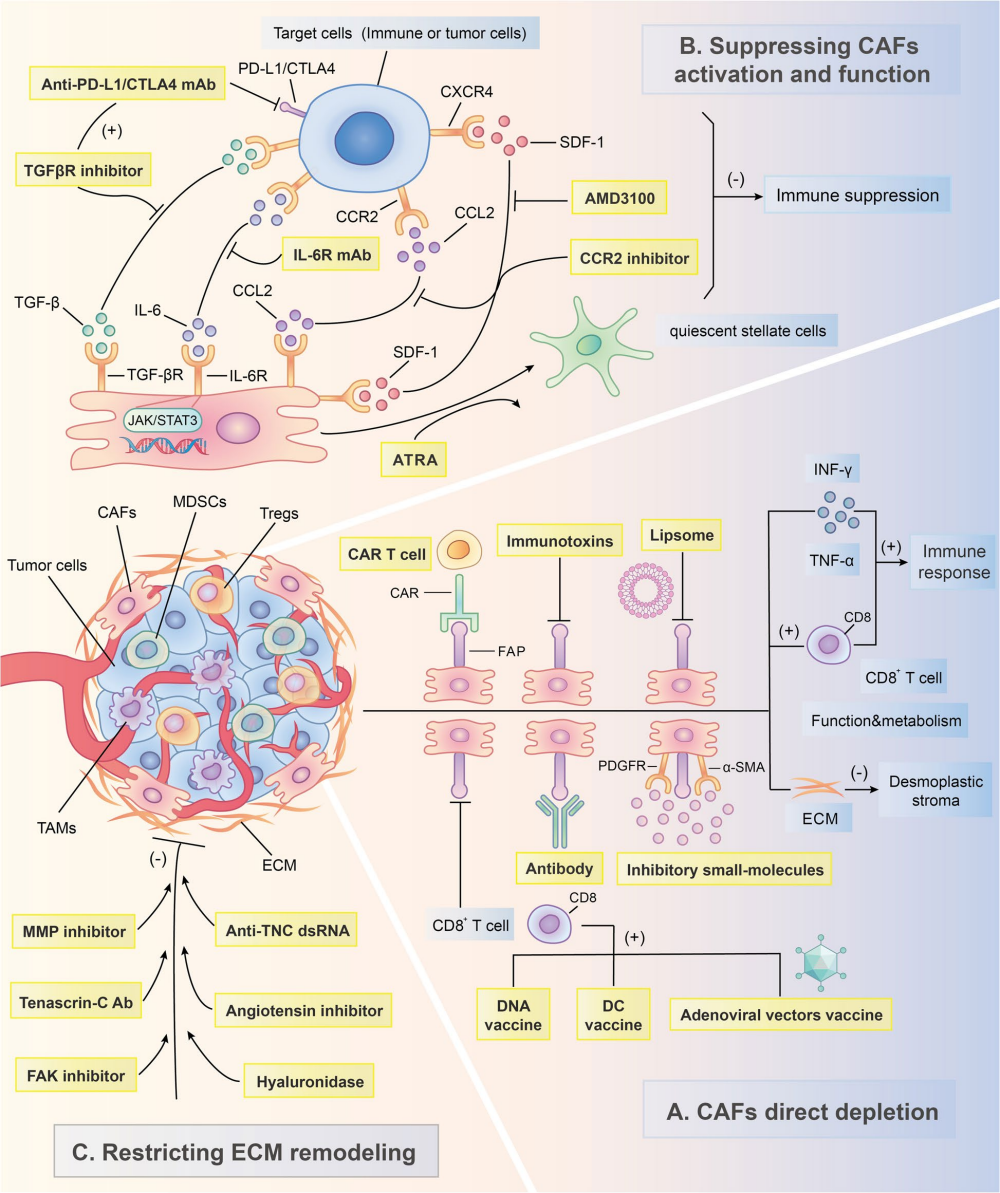
Major CAF-targeted immunotherapeutic strategies. There are three main approaches against cancer-associated fibroblasts (CAFs) and their associated molecules for immunotherapy: **A** Through the immunotherapies or transgenic technologies that targeting CAF markers such as fibroblast activation protein (FAP), alpha-smooth muscle actin (α-SMA) and platelet-derived growth factor receptors (PDGFR), CAFs can be directly depleted and consequently enhance immune response in the tumor microenvironment (TME); **B** CAF activation and function can be suppressed by inhibiting their crucial effector molecule or signaling pathways such as vitamin A, transforming growth factor-beta (TGF-β), interleukin-6 (IL-6) together with Janus kinase-signal transducer and activator of transcription 3 (JAK-STAT3) signaling pathway, C–C chemokine ligand 2 (CCL2)-C–C chemokine receptor (CCR2) signaling axis and C-X-C chemokine ligand 12 (CXCL12), thereby restricting the immune suppression induced by CAFs in the TME; **C** CAF-derived matrix proteins such as tenascin-C (TNC), hyaluronan (HA) and matrix metalloproteinases (MMPs) as well as related fibrosis-activated signaling pathways, like focal adhesion kinase (FAK) signaling pathways, are the ideal targets to effectively restrict extracellular matrix (ECM) remodeling

With in-depth research and an understanding of the immune response suppression driven by CAFs, these cells are becoming one of the most promising therapeutic targets for cancer intervention. In recent decades, the number of preclinical experiments that restore the anticancer immune response through CAF-targeted therapies has increased dramatically. Currently, there are three main strategies for CAF-based immunotherapy: direct CAF depletion, CAF activation and functional suppression along with CAF-induced ECM remodeling restriction (FIG. 3). Tables 3 & 4 briefly summarize the current therapeutic strategies against CAFs investigated in clinical and preclinical studies. In addition to immune checkpoint molecule-targeted inhibitors such as ipilimumab, pembrolizumab and nivolumab [294], CAF-specific therapies have been an essential complement to immunotherapies and have provided considerable clinical benefits for patients with tumors. However, due to the lack of specific markers for CAFs, as mentioned earlier [120], current CAF-targeting therapies have to address the intractable problem of how to improve the antitumor effect and decrease systematic side effects at the same time, and this issue might explain why only a few CAF-targeted therapies have been translated into the clinic. To discover more specific and efficient molecular targets for CAFs, further in-depth investigations on these cells are still required in the future.

**Table 3:** Multiple preclinical or clinical studies and related drugs for CAF-targeted depletion immunotherapy

Drugs	Classification	Combination therapy	therapeutic effects	Cancer models	Preclinical/Clinical	Refs
SynCon DNA vaccine	FAP-targeted DNA vaccine	Tumor antigen-specific DNA vaccine, Cyclo-phosphamide	Breaks immune tolerance and promotes antitumor immunity	Lung and breast cancer	Preclinical	[295–297]
DC-shA20-FAP-TRP2	FAP-targeted DC vaccine compound	Anti-CAFs therapies	Elicits broad-based T-cell responses and anticancer activities	Melanoma	Preclinical	[298, 299]
AdC68-mFAP vaccine	FAP-targeted adenoviral vectors vaccines	None	Enhances T-cell responses and inhibits tumor proliferation	Melanoma	Preclinical	[300]
FAP-specific CAR T cells	FAP-targeted adoptive T cell therapy	Gemcitabine	Enhances antitumor immune responses and restricts tumor proliferation, angio-genesis, ECM remodeling and chemoresistance	Pancreatic and lung cancer	Preclinical	[301]
ASGPAGPA-A12ADT/ DSGETGP-A12ADT	FAP-activated prodrugs	Thapsigargin	Enhances the specific antitumor effect of drugs with less systemic toxicity	Breast and prostate cancer	Preclinical	[302]
Val-boroPro (talabostat)	FAP-targeted inhibitory small-molecules	Cisplatin	Suppresses tumor growth and invasion and prolongs patients’ survival	Colorectal cancer, Melanoma	Phase II	[303, 304]
RG7386 (FAP-DR5 Antibody)	FAP-targeted inhibitory antibody	Irinotecan/ Doxorubicin	Induces cell-apoptosis and enhances antitumor immune responses	Lung, renal, colorectal, and breast cancer	Preclinical	[305]
αFAP-PE38	FAP-targeted inhibitory immunotoxins	Paclitaxel, Anti-CAF vaccine	Inhibits tumor angiogenesis and increases antitumor activities	Breast Cancer	Preclinical	[306, 307]
Bispecific scFv`FAP/CD105-IL liposomes	FAP-targeted compound liposomes	Doxorubicin/Trastuzumab	Enhances the cytotoxicity of Doxorubicin and cell interaction	Fibrosarcoma	Preclinical	[308]
Cellax (Docetaxel-conjugate nanoparticles)	α-SMA-targeted nanoparticles	None	Enhances anti-stromal effect and inhibits tumor metastasis and angiogenesis	PDAC and breast cancer	Preclinical	[309, 310]
Crenolanib	PDGFR-targeted inhibitor	None	Not available	Gastro-intestinal stromal tumor	Phase III	[311]

Diverse CAF-targeted depleting immunotherapeutic strategies in preclinical/clinical studies

*FAP* fibroblast activation protein, *DC* dendritic cell, *shA20* A20-specific shRNA, *TRP2* tyrosine-related protein 2, *AdC68* adenoviral vector of chimpanzee serotype 68, *CAR T cells* chimeric antigen receptor T cells, *CAFs* cancer-associated fibroblasts, *ECM* extracellular matrix, *DR5* death receptor 5, *PE38* a genetically engineered form of the Pseudomonas exotoxin, *scFv* single chain antibody fragment, *CD105* cluster of differentiation 105, *IL* interleukin, *α-SMA* alpha smooth muscle actin, *PDAC* pancreatic ductal adenocarcinoma, *PDGFR* platelet-derived growth factor receptor

**Table 4:** Diverse designed drugs that potentially target CAF-associated effector molecules, signaling pathways and matrix proteins

Drugs	Mechanisms	Combination therapy	therapeutic effects	Cancer models	Status	Refs
All-trans retinoic acid (ATRA)	Retinol levels restoration, PSC de-activation	None	Increases T-cell infiltration and Inhibits tumor growth and invasion	PDAC	Preclinical	[312]
Galunisertib	TGF-βR1 inhibition	Gemcitabine	Prolongs patients’ survival with minimal added toxicity	Pancreatic and hepatocellular cancer	Phase II	[313, 314]
Anti-CTLA4- TGF-βR2/Anti-PD-L1-TGF-βR2	TGF-βR2 and immune checkpoints inhibition	None	Decreases tumor-infiltrating Tregs and suppresses tumor progression	Breast cancer	Preclinical	[315]
Tocilizumab (monoclonal antibody)	IL-6-JAK/STAT3 signaling pathway inhibition	Carboplatin/Doxorubicin	Enhances antitumor immunity and provides survival benefits	Recurrent epithelial ovarian cancer	Phase I	[316]
CCX872	CCL2-CCR2 signaling axis inhibition	FOLFIRINOX (fluorouracil, leucovorin, irinotecan and oxaliplatin)	Restricts immune suppression and improves clinical prognosis	Pancreatic cancer	Phase I	[317, 318]
AMD3100	CCR4 inhibition	Anti-PD-L1 therapy	Promotes T-cell accumulation and eliminates cancer cells	Pancreatic cancer	Preclinical	[114]
F16-IL-2	Tenascin-C depletion and IL-2 delivery	Paclitaxel/ Doxorubicin	Enhances antitumor immunity and inhibits tumor growth	Breast cancer	Preclinical	[319]
VS-4718 (FAK inhibitor)	FAK-targeted inhibition	Anti-PD-1 therapy	Inhibits the infiltration of immuno-suppressive cells and improves survival	Non-small-cell lung cancer, mesothelioma and pancreatic neoplasms	Phase I	[320]
anti-TNC dsRNA (ATN-RNA)	Tenascin-C mRNA-targeted interference	Surgery	Prolongs patients’ survival and restricts tumor recurrence	Brain glioblastoma multiforme	Phase I	[321]
PEGPH20	Tumor stromal hyaluronan-targeted depletion	Gemcitabine and nab-paclitaxel	Prolongs patients’ survival with less systematic side effect	PDAC	Phase III	[322]
Losartan (angiotensin inhibitor)	Profibrotic signals inhibition	None	Facilitates drugs delivery and restricts ECM remodeling	Pancreatic and breast cancer	Preclinical	[323]

Diverse strategies in CAF-targeted immunotherapies that suppress CAF activation and function and restrict ECM remodeling

*CAF* cancer-associated fibroblast, *PSC* pancreatic stellate cell, *PDAC* pancreatic ductal adenocarcinoma, *TGF-βR1* transforming growth factor beta receptor 1, *TGF-βR2* transforming growth factor beta receptor 2, *CTLA-4* cytotoxic lymphocyte-associated antigen-4, *PD-L1* programmed death ligand 1, *IL-6* interleukin-6, *JAK* Janus kinase, *STAT3* signal transducer and activator of transcription 3, *CCX872* one of CCR2 antagonists, *CCL2* C–C chemokine ligand 2, *CCR2* C–C chemokine receptor 2, *AMD3100* one of CXCR4 antagonists, *CCR4* C–C chemokine receptor 4, *IL-2* interleukin-2, *FAK* focal adhesion kinase, *PD-1* programmed cell death protein 1, *TNC* tenascin-C, *PEGPH20* a PEGylated human recombinant PH20 hyaluronidase, *ECM* extracellular matrix

### Depleting CAFs directly by targeting surface markers

Direct CAF-depleting therapeutic strategies mainly depend on surface markers of CAFs, such as FAP, α-SMA and PDGFR. Therefore, CAF marker-based inhibitors are currently the major type of CAF-depleting therapies (Table 3). As one of the most viable CAF markers for potential clinical application, FAP has been prominent in recent studies of CAF-targeted therapy [324, 325]. The elimination of CAFs by FAP-targeting therapy can enhance the anticancer immune response mediated by high levels of certain inflammatory mediators, such as IFN-γ and TNF-α, and facilitate the toxic effects and metabolism of CD8 + T cells with a decreased desmoplastic stroma in the TME [224, 300, 301, 326]. Current FAP-targeting therapies mainly include diverse types of tumor vaccines and other immunotherapeutic approaches, such as adoptive T cell therapy, all of which can eliminate FAP + cells [327, 328].

FAP-based DNA vaccines are one of the principal types of cancer vaccines [315, 329]. The first DNA vaccine against the cancer stromal antigen FAP was developed by Loeffler et al. [330] in multidrug-resistant colon and breast carcinoma murine models. This vaccine is capable of eliminating CAFs by stimulating a CD8 + T cell-mediated immune response and further inhibit tumor growth and metastasis [330]. Recently, with advances in DNA vaccine studies, a novel type of vaccine termed the SynCon FAP DNA vaccine has been shown to not only disrupt immune tolerance and promote the antitumor immunity of both CD8 + and CD4 + T cells but also enhance the effects of other relevant tumor antigen-specific DNA vaccines [295]. Since the anticancer therapeutic effect of a single DNA vaccine targeting FAP is extremely limited [331], subsequent studies identified a novel therapeutic strategy that combines cyclophosphamide (CY) with a DNA vaccine that significantly increases the tumor inhibition rate by overcoming the tumor-stromal blockade and enhancing the nonspecific toxic effects of CY on tumor cells [296, 297].

In addition, DC vaccines are regarded as an effective strategy that induces a strong tumor immune response by replacing the role of impaired DCs in the TME [332]. Specifically, DC vaccines can enhance tumor antigen presentation by increasing costimulatory molecule and proinflammatory cytokine expression, thereby heightening cancer-specific T cell responses [333]. To improve the finite therapeutic effect of the previously established A20-silenced DC vaccine, Gottschalk et al. [298] developed a compound DC vaccine (DC-shA20-FAP-TRP2) that cotargets both tumor cells and FAP-positive CAFs. This vaccine was reported to elicit broad T cell responses and potent antitumor activity [298]. Moreover, when cooperating with other anti-CAF therapies, DC-based vaccines have been shown to reduce the level of TGF-β and consequently inhibit the migration of Treg cells into tumors [299]. Recently, studies have demonstrated that the fusion of DCs and CAFs contributes to a strong CTL response against CAFs, suggesting that it might be a potential method for improving the anticancer effect of current DC vaccine strategies [334]. Adenoviral vector vaccines are another type of FAP-targeting vaccine [335]. Similar to DNA vaccines, adenoviral vector vaccines such as the adenoviral vector of chimpanzee serotype 68 (AdC68)-mFAP vaccine can also induce T cell recruitment and enhance the function of melanoma-specific effector CD8 + T cells, thereby destroying FAP + stromal cells within the TME [300]. Additionally, other tumor vaccines contain whole-cell vaccines [312, 336] and peptide immunization vaccines [337].

FAP is also an important target for adoptive T cell therapy, especially for chimeric antigen receptor (CAR) therapy [338]. FAP-specific CAR T cells function to deplete most FAP + cells and restrict tumor stroma generation, along with promoting the uptake and antitumor effects of chemotherapeutic drugs, such as gemcitabine [301]. Notably, several studies have observed that the elimination of FAP + cells by CAR T cells causes severe side effects, such as significant bone marrow toxicity and cachexia [339, 340]. Considering that CAR T cells usually deplete FAP-overexpressing cells (e.g., CAFs) rather than normal cells with basal FAP levels, there might exist a different window of therapeutic opportunity for differential single-chain variable fragments (scFv) of CAR constructs [326]. In view of the possible toxicity of FAP-targeted adoptive T cell therapy, scientists try to develop prodrugs that are activated only by FAP through unique postprolyl endopeptidase activity, and these prodrugs have been proven to induce less systemic toxicity and have greater therapeutic potential [341, 342]. For instance, an in vivo and in vitro study in breast and prostate cancer illustrated that a FAP-activated prodrug contributed to the selective death of stromal cells and exerted a significant anticancer effect [302]. Finally, other FAP-targeting treatments, including FAP-inhibiting small molecules (talabostat and cisplatin) [303, 304], antibodies [305], immunotoxins [306, 307] and FAP-targeted liposomes [308, 343], also provide therapeutic benefits.

α-SMA has been identified as another surface marker of CAFs [324]. Current studies of therapies targeting α-SMA remain stagnant because of their dual effects on tumor progression. First, the depletion of α-SMA + CAFs was proven to suppress the metastasis of cancer cells as well as tumor angiogenesis in breast cancer and PDAC models [309, 310]. However, more importantly, targeting α-SMA was also reported to induce disease aggression and progression by enhancing the infiltration of CD3 + Foxp3 + Treg cells in the TME [310]. For other CAF markers, such as PDGFR, the associated clinical trials are still ongoing [311, 344].

As discussed before, neither FAP nor α-SMA is exclusively expressed on CAFs, suggesting that more highly selective markers are required to improve the precision of CAF-based therapies. Recently, Su et al. [104] identified two novel specific surface proteins for a CAF subpopulation (CD10 and GPR77), which might be promising targets for inhibiting tumorigenesis and tumor chemoresistance.

### Suppressing CAF activation and function by targeting associated effector molecules

Considering the crucial role of interactions between CAFs and other cells, particularly the crosstalk between CAFs and the TIME, in the immune suppression induction of the TME, it seems more feasible to restrict CAF activation and their interacting progress by targeting CAF-associated crucial effector molecules such as growth factors, cytokines and chemokines as well as signaling pathways (Table 4). For example, vitamin A deficiency is a main contributor to the activation of PSCs [43]. Therefore, by restoring retinol levels in PSCs, all-trans retinoic acid (ATRA) can reset them to the inactive state [43]. In a parallel study, ATRA treatment of a PDAC model exerted substantial antitumor effects, including remarkably increasing the numbers of CD8 + T cells in juxta-tumoral compartments and limiting tumor cell invasion [312]. TGF-β plays an important role in the activation of CAFs and the interaction between CAFs and immune cells, as previously described, indicating that TGF-β inhibition therapy might be capable of restoring impaired immune responses in the TME [30, 191, 213]. Currently, multiple preclinical and clinical studies of TGF-β-based immunotherapies are ongoing [192]. Galunisertib (LY21577299), for example, is a small-molecule inhibitor of transforming growth factor beta receptor 1 (TGF-βR1) with discernable cardiac toxicities rarely reported during treatment [345]. Phase II clinical trials for pancreatic cancer and hepatocellular carcinoma have exhibited the significant therapeutic activity of galunisertib against tumors, whether administered in combination with gemcitabine or as monotherapy [313, 314]. Additional reports have documented that the combination of a treatment targeting CAF-derived TGF-β with checkpoint inhibitors such as anti-PD-L1 antibodies exerts greater immunological effects on tumors than the respective monotherapies [346–348]. Therefore, Ravi et al. [349] attempted to engineer anti-CTLA4 or anti-PD-L1 antibodies fused with the TGF-βR2 extracellular domain, resulting in anti-CTLA4-TGF-βR2 and anti-PDL1-TGF-βR2 chimeras. Compared with ipilimumab (a type of anti-CTLA-4 antibody) monotherapy, the anti-CTLA4-TGF-βR2 molecule presents more effective at decreasing tumor-infiltrating Treg cells and suppressing tumor progression [315]. In addition, previous studies have demonstrated that IL-6 together with the JAK/STAT3 signaling pathway in the TME participate in processes that strongly suppress immune effector cell function and facilitate tumor progression induced by CAFs [122, 243, 350–352]. Tocilizumab, a humanized anti-IL-6R monoclonal antibody, has exhibited extensive antitumor and anti-chemoresistance effects on multiple cancer types in preclinical studies [353–355]. In a phase I clinical trial, high-dose tocilizumab was observed to stimulate CD8 + T cell activation and increase the levels of antitumor-associated effectors such as IFN-γ and TNF-α, thereby enhancing anticancer immunity [316]. Moreover, preclinical evidence indicates that therapy targeting IL-6/JAK/STAT3 signaling might also augment the antitumor efficacy of immune checkpoint-inhibiting monoclonal antibodies [356, 357]. Since the CCL2-CCR2 signaling axis plays an essential role in MDSC-induced immune suppression, therapies suppressing the CCL2-CCR2 signaling pathway might be effective in blunting MDSC immunoinhibitory effects [242]. Clinical trials have previously reported that CCR2 inhibition in combination with FOLFIRINOX (fluorouracil[5-FU], leucovorin, irinotecan and oxaliplatin) can significantly reduce the numbers of tumor-infiltrating macrophages and Treg cells while increasing the number of effector T lymphocytes in the TME, consequently enhancing antitumor immunity in pancreatic cancer [358]. Recently, a treatment combining CCX872 (a CCR2-specific antagonist) and FOLFIRINOX was reported to achieve a better therapeutic effect and clinical prognosis with less M-MDSC infiltration [317, 318]. Another essential chemokine, SDF-1 (also termed CXCL12), is also involved in the activation and immune suppression of CAFs. Via blocking the combination of SDF-1 and its receptor CXCR4, AMD3100 (a CXCR4 inhibitor) is able to rapidly promote the accumulation of T cells and effectively eliminate cancer cells by synergizing with an anti-PD-L1 antibody [114].

### Restricting CAF-induced ECM remodeling in the TME

CAF-targeted treatments are also being designed to block fibrosis progression, including therapies targeting fibrosis-activated signaling pathways and their fibrosis products (Table 4), which ultimately restrict CAF-induced ECM remodeling. The altered ECM after treatment partly alleviates the suppression of immune effector cell recruitment into tumors in the TME, thus enhancing anticancer immunity [281, 282, 359].

As mentioned before, the FAK signaling pathway is an important fibrosis-activated signaling pathway of CAFs involved in matrix stiffness and immune suppression [284]. A specific FAK inhibitor (VS-4718) was reported to inhibit immunosuppressive cell infiltration, such as TAMs, MDSCs and Treg cells, in the TME, and significantly improved overall survival (OS) in a PDAC model [284]. Moreover, as FAK inhibitors might heighten the antitumor effect of immune checkpoint inhibitors, associated phase I clinical trials have been set up to assess their therapy responses [320].

Therapies against CAF-derived ECM proteins, such as tenascin C (TNC), HA and MMPs, might also be capable of inhibiting desmoplastic reactions and consequently reducing the immunosuppressive effect of ECM on immune cells. The ECM protein TNC appears to be an appealing target for antitumor treatment due to its high expression in cancer tissues and functional association with tumor cell adhesion, migration, invasion and proliferation along with immune evasion [323]. Several antibodies have been established to specifically target TNC in order to improve the delivery of effector molecules into TNC-rich tumor tissue [360]. For example, the antibody F16 shows good specificity for TNC and is designed in complex with IL-2 to promote the recruitment of immune cells in the TME [361]. In a breast cancer model, when in combination with F16-IL-2, cytotoxic drugs such as paclitaxel or doxorubicin induce a more obvious restriction of tumor growth than chemotherapeutic agents alone [319]. Furthermore, anti-TNC dsRNA (ATN-RNA), which has sequence homology to TNC mRNA and is developed using RNA-based technologies, has produced substantial improvements in the clinical prognosis of patients with glioblastoma multiforme (GBM) [321]. Recent research in autophagy-deficient TN breast cancer revealed that TNC suppression sensitizes T cell-mediated cell killing and enhances the anticancer effects of single anti-PD1/PD-L1 therapy, indicating a potential therapeutic strategy that links TNC inhibitors and immune checkpoint blockade (ICB) for TN breast cancer [362]. Excessive tumor-stromal HA together with collagen usually results in substantial vessel compression, which blocks the delivery of peripheral immune cells and drugs into tumor vessels [363]. PEGPH20, a PEGylated human recombinant PH20 hyaluronidase, functions to deplete HA and then potentiate chemotherapeutic efficiency by improving vascular patency. Further clinical trials confirmed that PEGPH20 along with gemcitabine and nab-paclitaxel combination therapy could suppress tumor growth and significantly increase patient survival [322]. Moreover, losartan (an angiotensin inhibitor) also exhibits the ability to reduce the production of stromal collagen and HA by inhibiting TGF-β1, connective tissue growth factor (CTGF) and endothelin-1 (ET-1) profibrotic signals [323]. Finally, regarding MMP therapies, the disappointing antitumor effects of current MMP inhibitors have been gradually reported, but several novel types are being translated into early clinical trials [364].

## Challenges and directions

Considering a large number of CAF characteristics might change with the culture environment alteration (in vivo to in vitro), some questions have continuously arisen and need to be solved. First, to retain the CAF phenotype in in vitro culture as much as possible, researchers have tried various culture conditions and found that lower serum concentrations and matrices with more physiological mechanical properties might be preferable to keep the CAF original phenotype [87]. Recent studies have identified that several inhibitors of CAF activating molecules, such as TGF-β inhibitors, can effectively restrict the transformation of the CAF phenotype in vitro culture [365], which indicates that adding some CAF activator suppressors into in vivo culture medium might be a novel strategy to accurately preserve the in vivo phenotype of CAFs. Certainly, more in-depth studies are required to investigate more suitable in vitro culture conditions for CAFs.

Second, currently, single-cell transcriptome analyses have been a useful method to understand the characteristics and heterogeneity of CAFs. Aside from single-cell transcriptome analyses, researchers usually utilize immunoassay technology, such as high-quality antibodies against CAF marker proteins, to detect CAFs in tissue. However, due to the heterogeneity of CAFs, antibodies against certain CAF subpopulation markers require complex optimization, which hampers their adoption in laboratories. Recently, the technology of multiplexed mRNA probes has been rapidly developed, and thus accurate quantitative methods for the detection of CAFs, in the long term, are promising [87]. Further investigations should be performed to explore and develop more universal, stable, standardized and accurate quantitative methods for CAF detection in the future.

In addition, while multiple methods have been recently developed for the detection of CAF phenotype expression, as introduced above, such as specific antibodies, mRNA probes and transcriptome analyses, there is still a lack of a method to identify CAF phenotype changes in a timely and precise manner during the cultivation process.

Finally, it is necessary to deepen the understanding regarding the origins and subpopulations of CAFs, especially the time and stage heterogeneity of CAFs [366], by investigating CAFs in different experimental stages and different clinical stages. According to a previous study [367], for example, researchers can perform a longitudinal study of whole CAF populations in certain cancer animal models by utilizing whole transcriptome analysis in FACS-sorted fibroblasts from early to late stages or distinct pathological grades, and ultimately observe alterations in the CAF transcriptome and phenotype. Furthermore, a longitudinal study of certain fibroblast cell lines in different culture stages can also be conducted, including primary, early isolation, long-term passage and immortalization cells, through techniques such as single-cell transcriptome analyses.

Additionally, to date, the origins and subtypes of some CAFs, especially the anticancer subpopulation (rCAFs), are still unknown, and a deep understanding of rCAFs may become a future research direction. Moreover, the number of studies on CAF-immune cell interactions is far from sufficient, and most of the studies mentioned in our review have not completely illustrated the detailed cell-internal mechanisms by which CAFs affect immune cells. These factors should be considered in further subsequent investigations. Therefore, a thorough exploration of the crosstalk between CAFs and the TIME is required in the future to enable us to identify the basis of the impaired immune response induced by CAFs and might identify an essential method to restimulate the antitumor response which is distinct from strategies that directly restrict and eliminate cancer cells.

Furthermore, although an increasing number of CAF-targeting therapeutic strategies are being developed, the lack of more specific markers and the low number of large-scale randomized clinical trials are still two huge challenges facing CAF-targeting treatment.

## Conclusions

Since it acts as a crucial role in tumor initiation and progression in the TME, CAFs have received increasing attention in the past decade. CAF populations exhibit extensive heterogeneity in terms of cell origin and phenotype, which leads to their distinct behaviors during cancer development: most CAF subtypes (pCAFs) function as tumor facilitators; however, some other subtypes (rCAFs) exert tumor-inhibiting effects. Additionally, the intertransformation of several subpopulations partly indicates the plasticity of CAFs, while more investigations are needed to confirm this. Recently, studies have confirmed the importance of the interaction between CAFs and the immune microenvironment in the TME during tumor progression. Meanwhile, an increasing number of research regarding the effect of CAFs on the immune components of the TIME have gradually clarified the mechanisms by which CAFs orchestrate an immunosuppressive TME, and the results facilitate the translation of related CAF-based therapeutic targets into clinical trials.

In this review, we describe the interaction between CAFs and immune cells infiltrating the TME in detail and propose a possible immune inhibitory mechanism by which CAFs not only directly influence the activities of immune cells but also indirectly result in immune effector cell dysfunctions by upregulating immune checkpoint molecule expression on the cell surface and remodeling the ECM within the TME. By secreting various chemokines, cytokines and other effector molecules, CAFs directly inhibit immune cell-mediated antitumor immunity mainly through three main mechanisms, as listed below: (i) to drive the abnormal polarization or trans-differentiation of immune cells such as TAMs, TANs, MCs, DCs and T lymphocytes into certain procancerogenic cell subsets; (ii) to promote the activities of immune inhibitory cells, including M2-type TAMs, N2-type TANs, rDCs, Treg cells and MDSCs, in terms of their recruitment, infiltration, activation and immunosuppressive behaviors; and (iii) to reduce the cytotoxic activities and cytokine secretion of immune effector cells like NK cells and CTLs. Notably, some infiltrating immune cells, such as TAMs, TANs, MCs and DCs, are capable of enhancing the activation and function of CAFs. These interactions constitute immunoinhibitory loops that further heighten immune suppression in the TME. Moreover, CAFs have also been reported to modulate anticancer immunity through indirect means: (i) to upregulate the expression of immune checkpoint molecules such as PD-1/PD-L1 in both themselves and other cells in the TME to induce T cell dysfunction and immunologic tolerance; (ii) to degrade and remodel the ECM through the production of fibronectin, collagen and MMPs and the activation of FAK to restrict effector immune cell infiltration while increasing the recruitment of inhibitory immune cells, such as Tregs, MDSCs and TAMs, and consequently block the initiation of the immune response. Certainly, excess expression of immune checkpoints in CAFs and surrounding matrix deposition would in turn prolong CAF survival, stimulating their activation and maintaining their protumor properties. In view of the diverse immune suppressive effects of CAFs, current CAF-targeted therapeutic strategies that target CAF surface markers, associated effector molecules and their relevant signaling pathways along with restricted ECM remodeling have been developed to enhance antitumor immunity, which has produced considerable clinical benefits. More importantly, in combination with checkpoint blockade immunotherapies or chemotherapies, CAF-targeted treatment might hold promise for the treatment of tumors with a fibroblast-rich TME.

## Data Availability

Not applicable.

## Abbreviations

CAFs: Cancer-associated fibroblasts
TME: Tumor microenvironment
ECM: Extracellular matrix
TIME: Tumor immune microenvironment
α-SMA: Alpha smooth muscle actin
FAP: Fibroblast activation protein
FSP1: Fibroblast-specific protein 1
PDGFR: Platelet-derived growth factor receptor
CD: Cluster of differentiation
MMP: Matrix metalloproteinase
Treg: T regulatory
MDSCs: Myeloid-derived suppressor cells
IL: Interleukin
TGF: Transforming growth factor
HGF: Hepatocyte growth factor
PDGF: Platelet-derived growth factor
FGF-2: Fibroblast growth factor 2
SDF-1: Stromal-derived factor-1
ROS: Reactive oxygen species
PSCs: Pancreatic stellate cells
HSCs: Hepatic stellate cells
ISCs: Islet stellate cells
IGF-1: Insulin-like growth factor-1
MSCs: Mesenchymal stem cells
BMSCs: Bone marrow mesenchymal stem cells
EMT: Epithelial-mesenchymal transition
CXCL: C-X-C chemokine ligand
MZF1: Myeloid zinc finger 1
CCL: C–C chemokine ligand
ER: Endoplasmic reticulum
HASCs: Human adipose tissue-derived stem cells
ADFs: Adipocyte-derived fibroblasts
EndMT: Endothelial-to-mesenchymal transition
MMT: Mesothelial-mesenchymal transition
MMT: Monocyte-to-myofibroblast trans-differentiation
MAPK: P38-mitogen-activated protein kinase
JAK: Janus kinase
STAT3: Signal transducer and activator of transcription 3
DAMPs: Damage-associated molecular patterns
NLRP3: NOD-like receptor protein 3
HSF1: Heat shock factor 1
YAP: Yes-associated protein
TAZ: Tafazzin
DKK3: Dickkopf-3
LIF: Leukemia inhibitory factor
SHP-1: SH2-containing protein tyrosine phosphatase-1
PDAC: Pancreatic ductal adenocarcinoma
myCAFs: Myofibroblastic CAFs
iCAFs: Inflammatory CAFs
apCAFs: Antigen-presenting CAFs
MHC: Major histocompatibility complex
FB: Fibroblast population
POSTN: Periostin
MYH11: Myosin-11
PDPN: Podoplanin
CAV1: Caveolin 1
OSCC: Oral squamous cell carcinoma
HGSOC: High-grade serous ovarian cancer
pCAFs: Cancer-promoting CAFs
rCAFs: Cancer-restraining CAFs
BMP-4: Bone morphogenetic protein 4
EGFR: Epidermal growth factor receptor
TAMs: Tumor-associated macrophages
LPS: Lipopolysaccharide
TNF: Tumor necrosis factor
S100A4: S100 calcium binding protein A4
MCP-1: Monocyte chemotactic protein-1
Chi3L1: Chitinase 3-like 1
PD-1: Programmed cell death protein 1
M-CSF1: Macrophage colony-stimulating factor 1
ERα: Estrogen receptor alpha
SR-A: Class A scavenger receptors
TANs: Tumor-associated neutrophils
CLCF1: CAF-derived cardiotrophin-like cytokine factor 1
PD-L1: Programmed death ligand 1
CXCR: C-X-C chemokine receptor
GC-MSCs: Gastric cancer-mesenchymal stem cells
ERK1/2: Extracellular regulated protein kinases 1/2
MCs: Mast cells
VEGF: Vascular endothelial growth factor
SCF: Stem cell factor
NK: Natural killer
NKG: NK group
DNAM-1: DNAX accessory molecule 1
KIRs: Killer immunoglobulin-like receptors
PGE2: Prostaglandin E2
IDO: Indoleamine 2,3-dioxygenase
DAP12: DNAX-activation protein 12
IFN-γ: Interferon-γ
MICA/B: MHC class I chain-related gene A/B
PVR: Poliovirus receptor
DCs: Dendritic cells
rDCs: Regulatory DCs
TDO2: Tryptophan 2,3-dioxygenase
Th: Helper T
CTLs: Cytotoxic T lymphocytes
DPP4: Dipeptidyl peptidase IV
TSLP: Thymic stromal lymphopoietin
TLR8: Toll-like receptor 8
ICAM: Intercellular cell adhesion molecule
VCAM-1: Vascular cell adhesion molecule-1
HIC-5: Hydrogen peroxide inducible clone-5
TCR: T cell receptor
FAS: Factor associated suicide
FASL: Factor associated suicide ligand
PD-L2: Programmed death ligand 2
PMN-MDSCs: Polymorphonuclear MDSCs
M-MDSCs: Monocytic MDSCs
NO: Nitric oxide
LSCC: Lung squamous cell carcinoma
CCR: C–C chemokine receptor
TN: Triple-negative
GACs: Gastric adenocarcinomas
CTLA-4: Cytotoxic lymphocyte-associated antigen-4
TIM-3: Mucin-domain containing-3
LAG-3: Lymphocyte-activation gene-3
PI3K: Phosphatidylinositol 3-kinase
AKT(PKB): Protein kinase B
ERK5: Extracellular signal regulated kinase 5
ANLN: Anillin
DIAPH3: Diaphanous-related formin-3
SFKs: Src family kinases
FAK: Focal adhesion kinase
HA: Hyaluronan
CY: Cyclophosphamide
shA20: A20-specific shRNA
TRP2: Tyrosine-related protein 2
AdC68: Adenoviral vector of chimpanzee serotype 68
CAR: Chimeric antigen receptor
ScFv: Single-chain variable fragments
TGF-βR: Transforming growth factor beta receptor
TNC: Tenascin C
ATN-RNA: Anti-tenascin C dsRNA
ICB: Immune checkpoint blockade
CTGF: Connective tissue growth factor
ET-1: Endothelin-1
